# Spatial transcriptomics reveals novel genes during the remodelling of the embryonic human arterial valves

**DOI:** 10.1371/journal.pgen.1010777

**Published:** 2023-11-27

**Authors:** Rachel Queen, Moira Crosier, Lorraine Eley, Janet Kerwin, Jasmin E. Turner, Jianshi Yu, Ahlam Alqahtani, Tamilvendhan Dhanaseelan, Lynne Overman, Hannah Soetjoadi, Richard Baldock, Jonathan Coxhead, Veronika Boczonadi, Alex Laude, Simon J. Cockell, Maureen A. Kane, Steven Lisgo, Deborah J. Henderson

**Affiliations:** 1 Bioinformatics Support Unit, Faculty of Medical Sciences, Newcastle University, United Kingdom; 2 Human Developmental Biology Resource, Biosciences Institute, Faculty of Medical Sciences, Newcastle University, United Kingdom; 3 Biosciences Institute, Faculty of Medical Sciences, Newcastle University, United Kingdom; 4 Department of Pharmaceutical Sciences, University of Maryland School of Pharmacy, Baltimore, Maryland, United States of America; 5 MRC Human Genetics Unit, Institute of Genetics and Cancer, Edinburgh University, United Kingdom; 6 Genomics Core Facility, Biosciences Institute, Faculty of Medical Sciences, Newcastle University, United Kingdom; 7 Bioimaging Unit, Faculty of medical Sciences, Newcastle University, United Kingdom; 8 School of Biomedical, Nutritional and Sport Sciences, Faculty of Medical Sciences, Newcastle University, United Kingdom; Indiana University Purdue University at Indianapolis, UNITED STATES

## Abstract

Abnormalities of the arterial valves, including bicuspid aortic valve (BAV) are amongst the most common congenital defects and are a significant cause of morbidity as well as predisposition to disease in later life. Despite this, and compounded by their small size and relative inaccessibility, there is still much to understand about how the arterial valves form and remodel during embryogenesis, both at the morphological and genetic level. Here we set out to address this in human embryos, using Spatial Transcriptomics (ST). We show that ST can be used to investigate the transcriptome of the developing arterial valves, circumventing the problems of accurately dissecting out these tiny structures from the developing embryo. We show that the transcriptome of CS16 and CS19 arterial valves overlap considerably, despite being several days apart in terms of human gestation, and that expression data confirm that the great majority of the most differentially expressed genes are valve-specific. Moreover, we show that the transcriptome of the human arterial valves overlaps with that of mouse atrioventricular valves from a range of gestations, validating our dataset but also highlighting novel genes, including four that are not found in the mouse genome and have not previously been linked to valve development. Importantly, our data suggests that valve transcriptomes are under-represented when using commonly used databases to filter for genes important in cardiac development; this means that causative variants in valve-related genes may be excluded during filtering for genomic data analyses for, for example, BAV. Finally, we highlight “novel” pathways that likely play important roles in arterial valve development, showing that mouse knockouts of RBP1 have arterial valve defects. Thus, this study has confirmed the utility of ST for studies of the developing heart valves and broadens our knowledge of the genes and signalling pathways important in human valve development.

## Non-technical summary

Congenital heart defects, particularly those affecting the valves and septa of the heart, are very common. Despite this, few genes have been confirmed as disease-causing in human congenital heart (including valve) disease patients. Here we utilise a new technology that allows the identification of genes expressed in tissue slices from embryonic human heart valves and identify a set of genes that are human arterial valve-specific. We confirm by other methods that these genes are found in the arterial valves and highlight the relevance of the dataset by showing that mice that lack one of these genes, RBP1, have previously unidentified arterial valve defects. Using commonly used bioinformatic databases we show that these genes are not currently well represented. Thus, we confirm that new technologies can be used to study gene expression in tiny structures such as the developing heart valves and provide a new human embryonic valve dataset that can be used in future genomic studies of patients with congenital valve defects.

## Introduction

Abnormalities in the cardiac valves are amongst the most common congenital birth defects and cause significant morbidity throughout the lifespan. Over 1 in 100 of the population have a bicuspid aortic valve (BAV). Most are asymptomatic until adulthood when progressive fusion of the leaflet commissures causes clinically apparent aortic stenosis (AS). In addition, accelerated calcification and aneurysmal dilatation of the proximal aorta occurs in many BAV patients [1]. Fetal aortic stenosis is usually progressive and, at birth, may present in isolation as critical AS, or form part of complex lesions, for example, hypoplastic left heart syndrome [2]. Although in isolation BAV is often asymptomatic, it is frequently associated with more serious lesions and predisposes to severe cardiovascular disease in later life. For this reason, it has received considerable attention in recent years.

Although a number of large-scale genomic studies have been carried out to look for disease genes in patients with congenital heart defects (CHD) [3–5] and more specifically BAV [6–14], few genes have been identified, and even fewer gene variants have so far been verified as specifically causing BAV. In those cases where genes have been suggested to be causative for BAV, the whittling down of huge numbers of patient variants to a handful of gene candidates has been carried out by reference to datasets of genes known to be important in cardiac development–heavily reliant on developmental genetic studies in animal models (for example [6,7,13,14]). Thus, identification of genes as being expressed in the developing mouse heart, followed by functional analysis of these genes in animal models, remains the cornerstone of detection of human CHD and BAV causative genes. Arguably, however, the absence of valve-specific gene expression datasets is hampering this approach and explains, at least in part, the difficulties experienced in confirming gene variants as causative for BAV.

Although aortic valve disease is an area of major clinical importance, most of what we know is derived from analysis of the atrioventricular valves. Only recently have studies focussed specifically on the differential development of the arterial valves [15–19]. Consequently, while our knowledge of the formation of the cushions, the precursors for the mature valve leaflets is relatively well established, comparatively little is known about how these bulky structures remodel into sculpted valve leaflets. Studies in zebrafish have suggested that haemodynamic forces resulting from blood flow through the heart are critical for this remodelling process [20–22], but how they integrate with intrinsic, genetically regulated, signalling cascades remains unclear. Notably, there remains a specific lack in our current understanding about the development of the human arterial valve leaflets [23–25], although it is generally assumed that the morphogenetic processes will be similar to those implicated from the study of animal models. Although this is likely correct, there is some evidence for different patterns of aortic valve anomalies in mouse and human, indicating that the reliance on particular processes or susceptibility to pathology may vary between the two species (discussed in [19]).

Single cell transcriptomics (sc-RNASeq) has become commonplace over recent years, with increasing evidence that this approach can bring new insights to normal physiology and disease pathology [26]. Here we utilised a spatial transcriptomic approach [27–29] to derive data about gene expression in the arterial valves of the human embryo, during the sculpting phases of their development. We have been able to validate these gene sets by comparison with sc-RNAseq and in some cases spatial transcriptomic datasets from other studies of fetal and postnatal valves, as well as by confirmation through RNA in situ expression experiments in mouse and human embryos. We developed bioinformatic approaches for transcriptomic analysis, which were used to identify a number of genes that have not been previously linked to valve development and are strongly expressed in the remodelling valve leaflets. Examination of the mouse model for one of these, RBP1, shows previously undescribed defects in the great arteries and valve anomalies in mutant animals, highlighting that these genes are good candidate genes for causing BAV. Through the use of specialised spatial analysis methodologies, we show ST datasets have the ability to identify previously unknown developmentally important genes. We have shown that ST can be used as a powerful technique for novel gene discovery in the developing human embryo.

## Results

### Optimisation of spatial transcriptomics for analysis of developing human heart valves

There have been few transcriptomic analyses of developing heart valves, particularly in the human embryo. To circumvent the problem of dissecting out these tiny structures in this scarce material, we utilised ST technology to bioinformatically distinguish the forming valves from the surrounding myocardial tissue.

**Fig 1:**
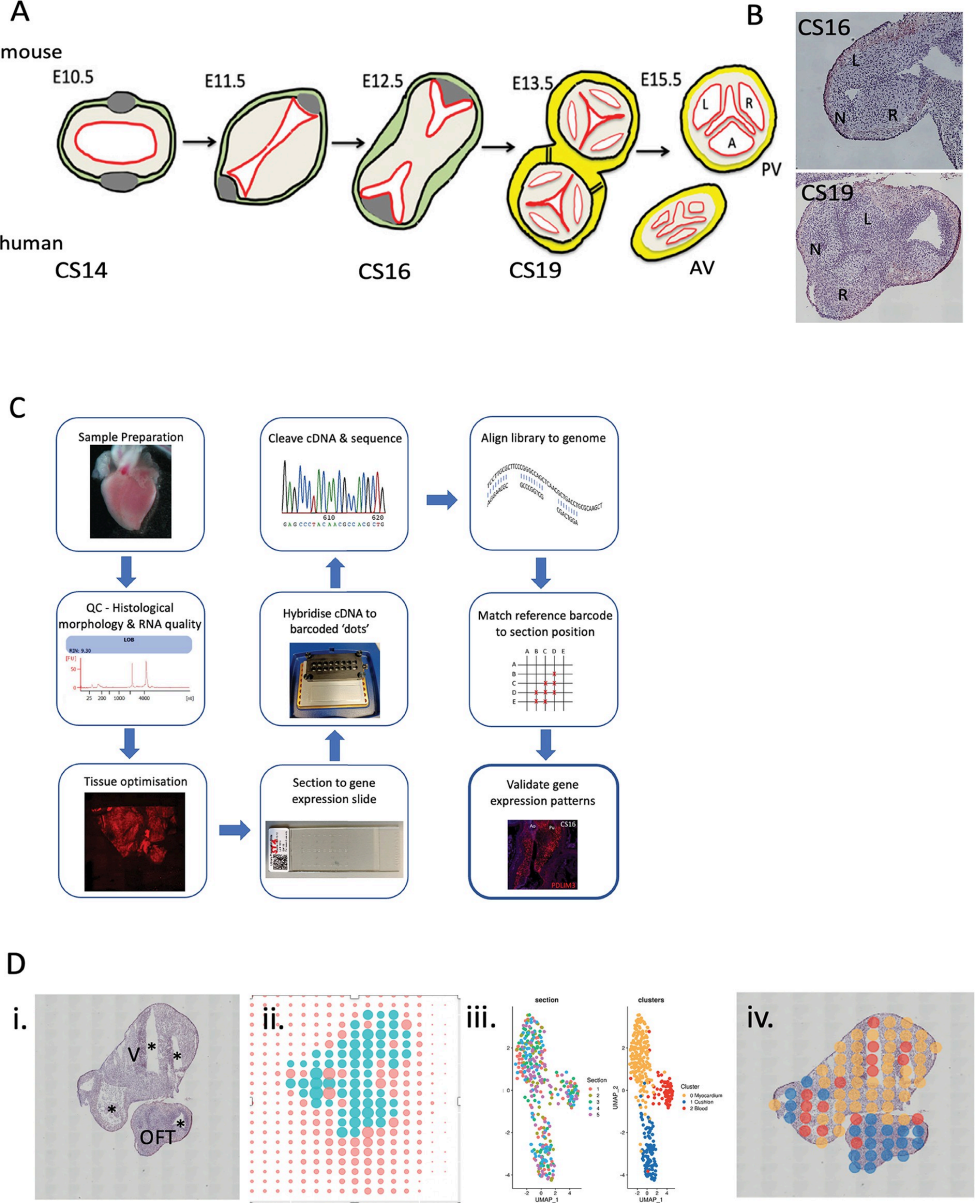
Overview of ST process. **A)** Cartoon showing stages of arterial valve development in mouse with human equivalents. **B)** H&E -stained sections of CS16 and CS19 human arterial valves used for analysis. **C)** workflow of ST including quality control steps. **D)** Example of output of ST for CS19 embryo: i) H&E section; ii) position of spots on hybridisation slide with turquoise spots covering tissue section and orange spots covering blank slide; iii) the first graph shows that data from each of the 5 slides (each with a different colour) from the CS19 embryo slide overlaps following clustering, whilst the second graph shows the three clusters (yellow, red, blue) obtained; iv) three clusters mapped back onto H&E sections. AV = aortic valve; L = left leaflet; N = non-coronary leaflet; OFT = outflow tract; PV = pulmonary valve; R = right leaflet; V = ventricle; * lumen of chambers and/or outflow tract.

Dissected and cryo-embedded chromosomally normal CS16 (approximately 37 post conception days) and CS19 (approximately 48 post conception days) hearts, both from female embryos, were chosen for analysis, as these map to the period of development when the arterial valves are beginning to remodel from bulky cushions to more sculpted valve leaflets—a time frame where relatively little is known (Fig 1A). Hearts were sectioned in a cross-sectional plane through the aortic and pulmonary valve leaflets so that each of the three leaflets would be included in the analysis. H&E staining confirmed that the three valve leaflets could be readily distinguished in these hearts (Figs 1B and S1).

An overview of the adopted experimental design is outlined in Fig 1C and follows a previously described method [30] with the addition of quality control checks prior to the beginning of the workflow and following bioinformatics analysis, as well as supplementary data analysis which involved positioning of the transcripts to digitally annotated anatomical regions. On average there were more than 6400 counts and 2200 genes found in each spot at CS16, and 17000 counts and 3700 genes per spot at CS19. Cluster analysis of the technical replicates from each time point individually resulted in three clusters when a resolution of 0.8 was used (Fig 1D). Clustering of spots was not section-specific and spots from all sections at a particular stage were found throughout the dataset (Fig 1D). This indicates that the ST is reproducible and shows that clusters identified are not driven by technical factors in the data.

### The transcriptomes of CS16 and CS19 arterial valves are comparable

**Fig 2:**
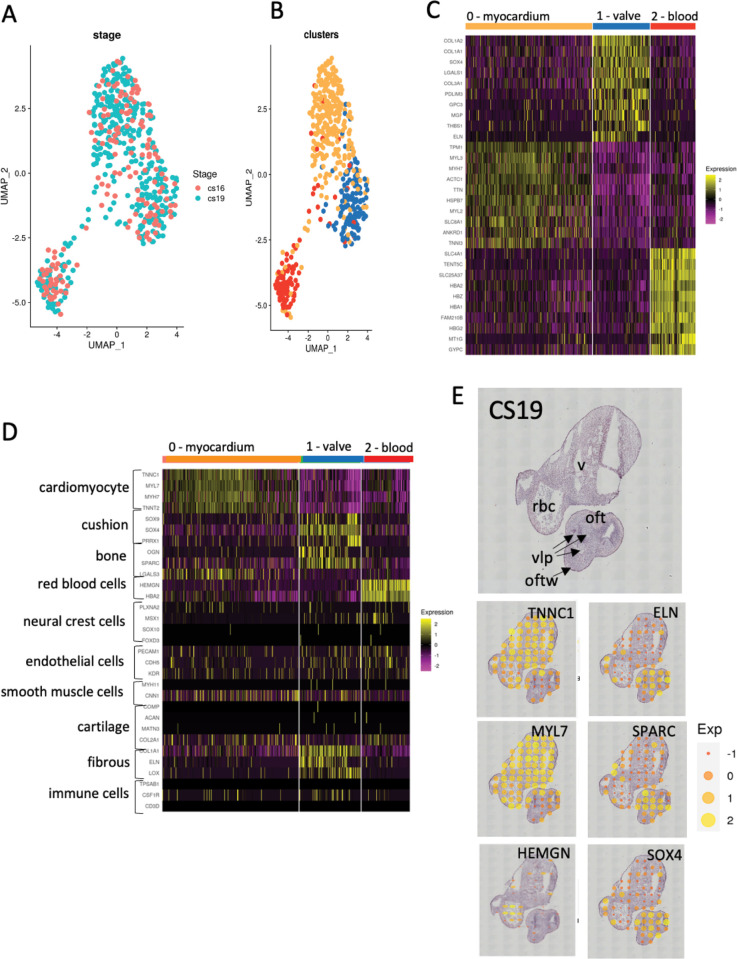
Cluster analysis. **A)** UMAP projection showing overlap of data between sections from the CS16 (orange dots) and CS19 (turquoise dots) embryos. **B)** UMAP projection showing clustering of combined CS16 and CS19 data, again showing three clusters (red, blue and orange dots). **C)** Heat map showing top 10 genes in each cluster, highlighting the gene expression differences between the three clusters. At this stage, based on the expressed genes, putative tissue types can be proposed as myocardium (orange), cushion (blue) and blood (red). **D)** Perfect marker analysis using recognised markers for specific cardiac cell types confirms the orange cluster as myocardium, the red cluster as blood cells, and shows that the putative cushion cluster has characteristics of cushions, bone and fibrous tissue, and thus should be better named as “valve” as this implies the entire structure, not just the developing leaflets. **E)** Mapping “perfect markers” back to the H&E sections shows that they localise to the expected areas (compare to Fig 1D). The H&E section shows the identity of the tissues and structures contained within the section. oft = outflow tract, oftw = outflow tract wall, rbc = red blood cells (in lumen), v = ventricular myocardium, vlp = valve leaflet primordia.

To further confirm that the transcriptomic data obtained were biologically reproducible, we compared the datasets obtained at CS16 with that obtained at CS19 and visualised them on a UMAP. These analyses showed that, similar to Asp et al [31], the two datasets overlapped, with no obvious separation between them (Fig 2A). Despite the difference in developmental maturity between the two samples, their transcriptomes were highly comparable. This allowed the two samples to act as biological controls for one another and for this reason, further analyses were carried out using a combined CS16/19 dataset.

The differentially expressed genes (DEGs) at CS16/19 fell into three clusters (Fig 2B), with heatmaps of the most highly DEGs (differentially expressed between the three clusters) confirming clear separation (Fig 2C). “Perfect Marker” analysis, using marker genes identified in previous studies as being characteristic of specific differentiated cell types in the heart (for example [28,31,32]), confirmed two of the three clusters as cardiomyocytes (cluster 0) and blood cells (cluster 2). The relevant “Perfect Marker” genes mapped back to the expected regions of the histological sections at both time points (Figs 2E and S2). The third putative cluster (cluster 1) was more complex than the others, with genes characteristic of endocardial cushion tissue, bone and fibrous tissue. As endocardial cushions (the precursors of the valve leaflets) have been described previously to share many markers with developing bone ([33]; see below) and the walls surrounding the maturing cushions/valves become progressively fibrous [34] this cluster likely corresponds to the developing valve complex. Moreover, the relevant marker genes mapped back to the arterial valve region on the tissue sections (Figs 2E and S2). Interestingly, neural crest cell marker genes were not abundant in the tissue, suggesting that these progenitor cells had differentiated by CS16, nor were genes typically expressed in smooth muscle cells or cartilage. Typical endothelial cell genes were evenly distributed between the three clusters, reflecting the abundance of this cell type in all tissues (Figs 2D and S2).

### Novel arterial valve-specific genes in human arterial valves

**Fig 3:**
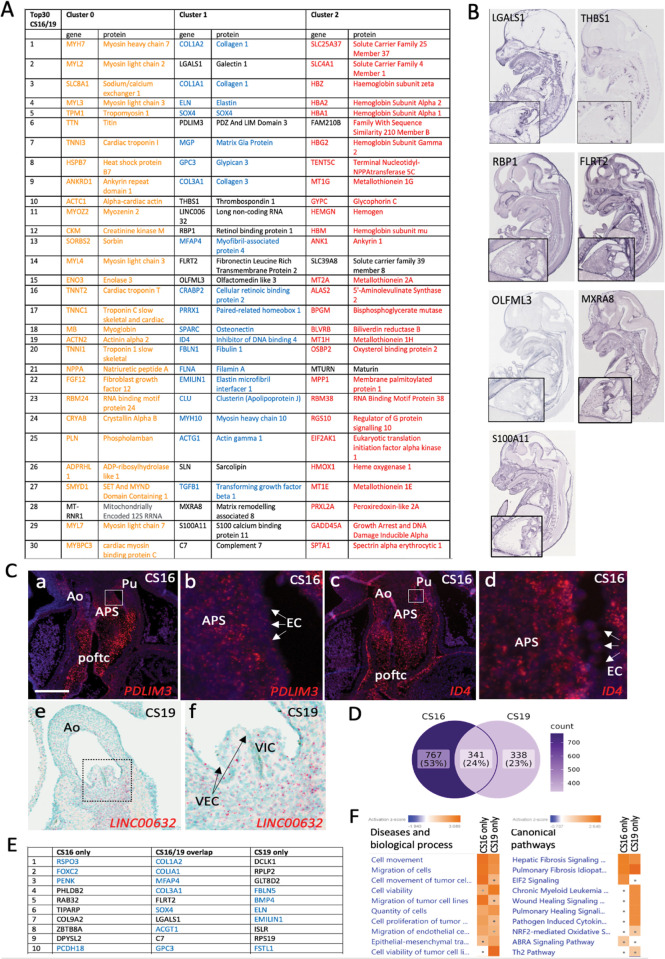
Identification of cluster genes. **A)** Top 30 DEGs in each of the three clusters. In each case, the coloured genes are already documented to be expressed in the proposed cluster tissue, whereas genes in black font were not identified as being expressed in the relevant tissue by literature search. **B)** Expression of novel Cluster 1 (valve) genes in the developing E14.5 mouse heart (GenePaint images) Higher power images of the heart are included for each gene in the bottom left corner of each image. **C)** PDLIM3, ID4 and LINC00632 transcripts (red dots in all cases) are all found in the CS16/CS19 human arterial valves. White box denotes the area covered by the high-power image for each gene (b,d,f). PDLIM3 and ID4 are expressed at CS16 in the cushion mesenchyme, although ID4 is more restricted to the distal region close to where the valves will form. Neither are expressed at high level in the endocardium of the forming valve region (arrows). LINC00632 is also expressed in the valve leaflets and supporting mesenchyme at CS19 and is not expressed in the developing valve endocardial cells (arrows). **D)** Venn diagram showing the distribution of valve genes that are specific to CS16, expressed at both CS16 and CS19, or specific to CS19. The numbers reflect the number of genes in each category. **E)** Top 10 valve genes that are specific to CS16, expressed at both CS16 and CS19, or specific to CS19. Blue colour denotes genes that are already known to be expressed in the developing or mature valve. All of the genes in the CS16/19 overlap are also found in the top 30 most highly DEGs when the two datasets are integrated and analysed together (compare with (A). **F)** IPA shows that most of the genes fall into similar pathways for disease and biological processes. There are more differences for canonical signalling pathways, although the top two are related to fibrosis and are shared. The dot in some boxes denotes the result was not statistically significant. Ao = aortic valve; APS = aortopulmonary septum; EC = endocardium; poftc = proximal outflow tract cushions; Pu = pulmonary valve; VEC = valve endocardial cells; VIC = valve interstitial cells. Scale bar in C = 300μm in a,c, 40μm in b,d, 200μm in e, 66μm in f.

The top 30 DEGs in the combined CS16/CS19 dataset, for each of the three clusters, are shown in Fig 3A (For full gene lists see S1–S3 Tables; GEO reference: GSE244723). Genes that have been previously identified as being strongly expressed in cardiomyocytes, cushions/valves or blood are colour coded as for the cluster diagrams, whereas those previously not known to be expressed in the relevant tissues (although they may be known to be expressed in the heart in general) are denoted in black. Analysis of the genes in these clusters supports the idea that cluster 0 represents cardiomyocytes, with several members of the myosin light and heavy chain, troponin and tropomyosin families most highly expressed; 29 genes in the top 30 had previously been shown to be highly expressed in cardiomyocytes. In the case of the putative “cushion/valve” cluster (cluster 1), the most highly expressed genes have previously been associated with the developing arterial wall that surrounds the developing valve complex (including COL1A1, COLIA2, COL3A1 and ELN), as well as genes associated with the developing cushions such as SOX4. This supports the findings of the “perfect marker” analysis, which showed the presence of fibrous markers known to be abundant within the arterial wall associated with this cluster. It would therefore seem more appropriate to characterise this as a “valve” cluster as this more general term would include the cushion-derived leaflets as well as the supporting arterial structures. Cluster 2 was confirmed as blood cells within the lumen of the heart and includes an abundance of genes known to be expressed in erythrocytes including multiple haemoglobin isoforms (Fig 3A).

We focussed on the valve cluster which was the main interest of our study. The majority of the top 30 DEGs had been previously associated with valve tissues, with analysis in the GenePaint mouse gene expression database (gp3.mpg.de) revealing remarkable valve-specificity (Figs 3B and S3). However, for 11/30 of the genes: LGALS1, PDLIM3, THBS1, LINC00632, RBP1, FLRT2, OLFML3, SLN, MXRA8, S100A11 and C7 there were, to the best of our knowledge, no published papers describing functions within the developing outflow tract, including the arterial valves. Of these “novel” valve genes, GenePaint showed that LGALS1, THBS1, RBP1, FLRT2, OLFML3, MXRA8 and S100A11 were all expressed in the arterial valve region in the E14.5 mouse, with no information available for PDLIM3 and LINC00632 (Fig 3B). SLN and C7 were represented in GenePaint but there was no clear localisation to the arterial valves (S3 Fig). We therefore carried out RNAScope and BaseScope gene expression analysis to investigate the expression pattern of PDLIM3 and LINC00632 respectively, as well as ID4 which is known to be expressed in the heart but lacked relevant expression data in GenePaint. Remarkably, all three genes localised to the arterial roots at CS16 and CS19 (Figs 3C and S3B). Thus, the ST method we utilised has proven useful for identifying genes previously unknown to be involved in arterial valve development.

Although the UMAP suggested that the CS16 and CS19 could be combined, we were interested to see whether there were differences between the CS16 and CS19 valve datasets. To address this, bioinformatic analysis was carried out on the separate datasets. Of the almost 1500 genes that were expressed in the arterial valves at CS16 and CS19, 24% were found at both time points, with 53% found solely at CS16 (Fig 3D). Twenty of the top 30 genes expressed at both time points were already known to be linked to the cardiac valves (Figs 3E and S4), whereas at CS16 and CS19, 15/30 and 11/30, respectively, were already known to be valve-related. Interestingly, particularly at CS16, several of the “novel” genes could not be localised to the valve region (GenePaint data), suggesting that the separated data may not be so robust (S4 Fig). Unsurprisingly, the majority of the shared genes were also top hits when the data was analysed as a combined dataset (compare Figs 3A and S4). Ingenuity Pathway Analysis comparing the two datasets showed that the top 10 associated Diseases and Biological Functions were very similar between the two datasets, with the majority associated with either cell movement or cell turnover (Fig 3F). The top two Canonical Pathways (both fibrosis-related) were shared between the two datasets, with pathways linked to wound healing (also linked to fibrosis) more activated in the CS19 dataset compared to CS16 (Fig 3F). Thus, it was concluded that although there were differences between the two datasets, the considerable similarities between them, together with the benefit of biological replication, confirmed the advantages of using the combined dataset.

### The developing human arterial valve transcriptome is similar to mouse

**Fig 4:**
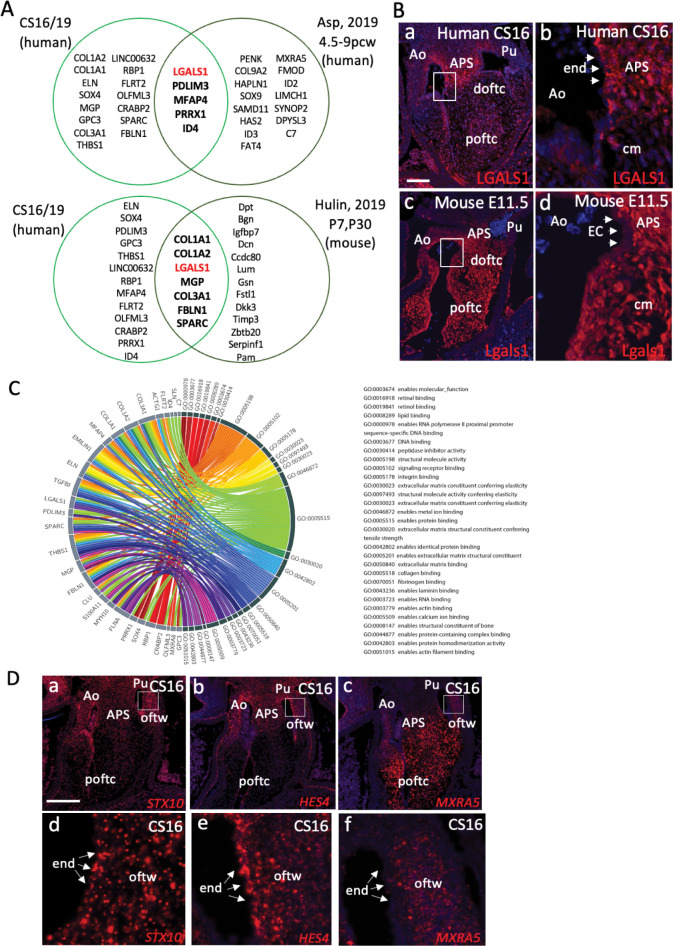
Comparison of “valve” genes to published ST/single cell RNASeq datasets. **A)** Comparison of the top 20 genes in our valve cluster with the human embryonic “atrioventricular mesenchyme and valve” ST cluster from Asp et al (2019) reveals 5 shared genes. Similarly, comparison of our top 30 valve cluster genes with scRNASeq “VIC” cluster data from P7 and P30 mouse atrioventricular and arterial valves (Hulin et al, 2019) reveals 7 genes in common. Notably, LGALS1, which has not previously been reported to be specific to the arterial valves, is found in all 3 datasets. **B)** Immunohistochemistry for LGALS1 in human and mouse embryonic hearts. White boxes denote the area covered by the high-power images (b,d). LGALS1 is expressed at high level in the developing aorto-pulmonary septum and at lower level in the mesenchyme of the distal and proximal outflow tract cushions of the human CS16 heart. In the E11.5 mouse heart, Lgals1 is found at high level in the aortopulmonary septum and throughout the distal and proximal cushions. LGALS1/Lgals1 is found at only low level in the endocardium in both species (arrowheads). **C)** Circos plot showing the top 30 genes in our valve cluster mapped to GO terms. **D)** STX10, HES4 and MRXA5, none of which are found in the mouse genome, are expressed (red dots) in the developing outflow tract at CS16. White boxes denote the area covered by the high-power images (d,e,f). MXRA5 is expressed at high level in the aortopulmonary septum (APS) and the proximal cushions, whereas HES4 is restricted to the APS and STX10 is found at lower level in both tissues. STX10 and HES4 are strongly expressed in the forming walls of the arterial roots, whereas MXRA5 is found only at low level in this tissue. Whereas all three genes are found in the walls of the forming arterial roots, only STX10 and HES4 are also found in the endocardium in this region. Ao = aortic valve; APS = aortopulmonary septum; cm = cushion mesenchyme; doftc = distal outflow tract cushions; end = endocardium; oftw = outflow tract wall; poftc = proximal outflow tract cushions; Pu = pulmonary valve. Scale bar in B = 100μm in a,c, 20μm in b,d. Scale bar in D = 300μm in a-c, 40μm in d-f.

We compared our data with other published datasets; it should be noted that the available data varied between studies and this influenced the depth of the comparisons that could be made. Our aim here was not to suggest that our dataset replicates these other non-identical datasets, but that there are enough genes in common to support the idea that our data is scientifically sound. Whilst there were no datasets that were directly parallel either in human or mouse (where E12.5-E13.5 best matches the CS16/19 samples we used), a comparison with the top 250 DEGs between E11 mouse AV/OFT compared to ventricle [35] and our top 250 revealed a 20% overlap (S4 Table). Better overlap was obtained with a spatial transcriptomics dataset from 4.5–9 weeks post conception human hearts [31] where the figure was closer to 30% for a comparison between the AV cushions/valves cluster for the top 250 genes. Comparing the top 20 genes, five genes (25%; LGALS1, PDLIM3, MFAP4, PRRX1 and ID4) were common to both datasets Fig 4A). Comparison with the outflow tract/large vessel cluster from the Asp dataset revealed 70/250 overlapping genes and two (ELN, CRABP2) were shared between the top 20 from both datasets (S4 Table). We next compared our data with single cell RNAseq data from aortic/mitral valves from postnatal day (P) 7 and P30 mouse [36]. Although there was little overlap between our dataset and valve endothelial cells, valve immune cells or melanocytes, there was remarkable overlap with the most highly expressed genes in valve interstitial cells (VIC) (Fig 4A), which made up more than 75% of the cells in their dataset. COL1A1, COL1A2, LGALS1, MGP, COL3A1, FBLN1 and SPARC were in the top 20 DEGs in both datasets. Of interest, one gene, LGALS1 was found in the top 20 DEGs in ours, the Asp (AV mesenchyme and valves; [31]) and the Hulin [35] datasets. Expression analysis in mouse and human using an LGALS1-specific antibody showed that it was expressed in the developing cushions in both species at E11.5/CS16. However, whereas Lgals1 was expressed at high level throughout the outflow cushion mesenchyme (interstitial cells) and forming aorto-pulmonary septum in the mouse, it was found at lower level in the cushion mesenchyme in CS16 human embryos, although there was high level expression in the aorto-pulmonary septum. In both species there was limited expression in endocardial cells (Fig 4B). Thus, our dataset validates previous microarray/RNASeq experiments carried out with mouse and human valve tissue, adding a significant number of “novel” genes–including LGALS1—not previously identified as being important in developing arterial valve tissue.

We analysed the top 30 genes identified in our combined CS16/19 valve dataset in GORILLA (http://cbl-gorilla.cs.technion.ac.il/), which allowed us to determine the molecular function of the genes. The most common functions were “enables protein binding”, “structural molecule activity” and “enables extracellular matrix structural constituent” (Fig 4C). The three transcriptional regulators in our top 30 most highly expressed genes in the combined CS16/19 dataset were SOX4, PRRX1 and ID4 (Figs 3A and 4C). We also analysed the top 250 DEGs identified in our combined CS16/19 valve dataset in PANTHER (http://www.pantherdb.org), which allowed us to determine the protein class for the genes. The most abundant categories were cytoskeletal proteins, extracellular matrix molecules, gene specific transcriptional regulators and metabolite interconversion enzymes (S4 Fig). We also looked at the biological processes implicated by our top 250 valves genes, again using PANTHER. This suggested that genes involved in “cellular processes”, “biological regulation” and “metabolic processes” were most abundant (Figs 4C and S5).

Of particular interest, four genes in the top 250 (STX10, HES4 and MXRA5, as well as LINC00632; described earlier) were not found in the mouse genome. RNAscope analysis revealed that like LINC00632 (Fig 3C), STX10, HES4 and MXRA5 were all expressed in the developing valve complex (Fig 4D). However, whereas STX10 and HES4 appeared to be largely restricted to the walls of the forming arterial roots, MXRA5 was found in the endocardial cushions that will give rise to the valve leaflets. Thus, we have identified genes highly expressed in the developing human valve complex that would not be picked up in transcriptomic studies in mouse embryos.

### Pathway analysis suggests valves genes under-representation of valve transcriptomes in IPA

**Fig 5:**
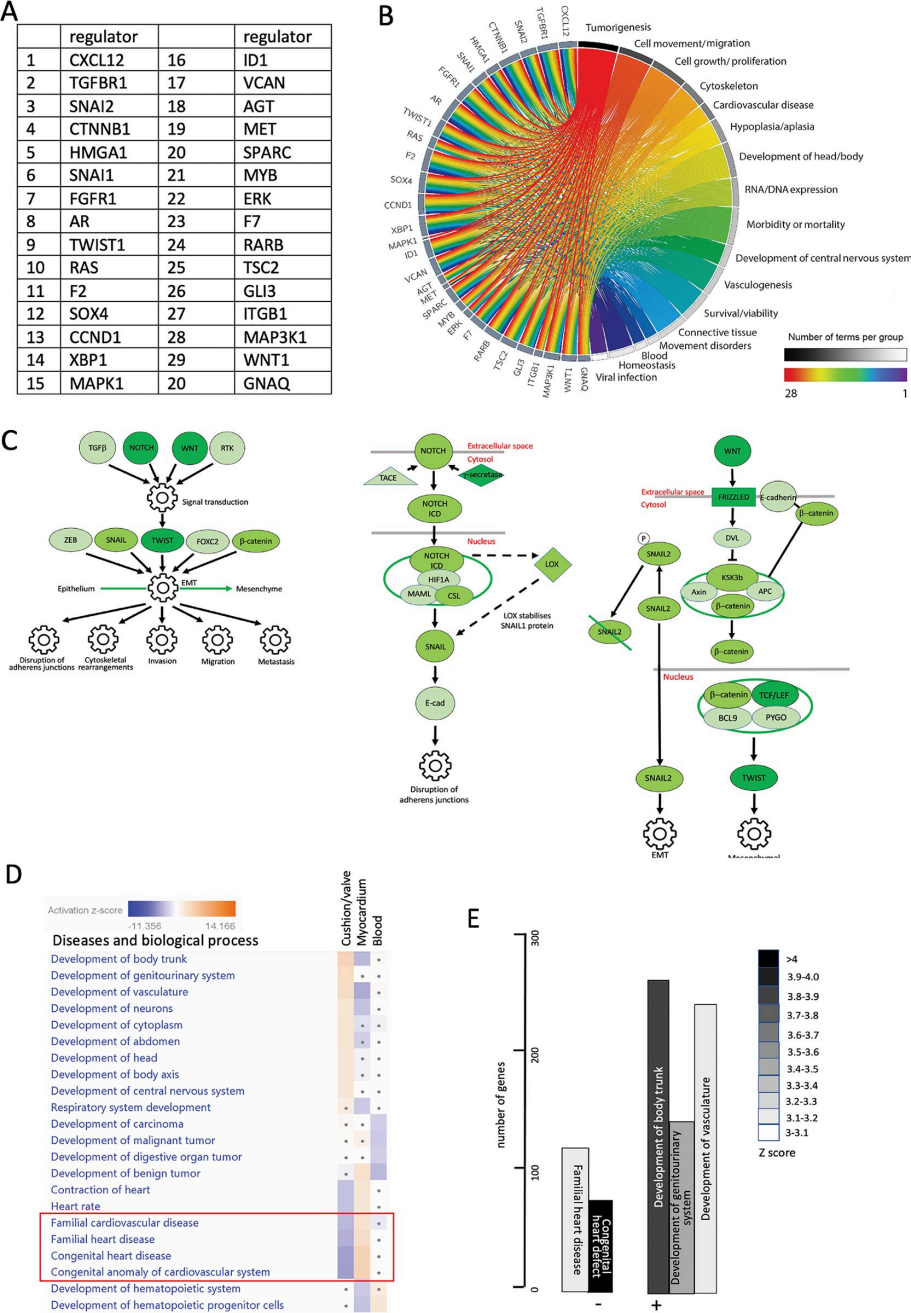
Functional associations of valve cluster genes generated by filtered IPA analysis. **A)** Top 20 regulators of genes in the valve cluster. **B)** Circos plot linking valve regulators to cellular processes. The strongest associations relate to cell movement and growth/proliferation but cardiovascular disease is also a string association for this group of valve regulatory genes. **C)** Following filtering for the terms “cardiac” and “development” regulation of EMT was the only pathway significantly upregulated in the valve dataset compared to the myocardial and blood clusters. Notch and Wnt were the likely upstream regulators. Pathways highlight the genes in the Notch and Wnt pathways that are positively associated regulators of EMT for the valve cluster (dark green genes are the most differentially upregulated, with mid green and light green progressively less so). **D,E)** IPA pathway analysis shows that for disease and biological processes, developmental terms such as development of trunk, genitourinary system, vasculature and neurons were strongly associated with the valve cluster. Negative associations were also found, the most striking being those to familial heart/cardiovascular disease, and congenital heart/cardiovascular disease/anomaly. These terms were positively associated with the myocardial cluster dataset (red box).

We carried out Ingenuity pathway analysis (IPA) on the valve cluster data to further link our data into pathways and processes. We first looked at the predicted upstream regulators associated with our dataset (Figs 5A and S6). This suggested CXCL12 and TGFBR1, both of which are implicated in cushion/valve development [37,38] as the most important regulators. Several Epithelial Mesenchymal Transition (EMT) regulators including SNAI2, CTTNB1, SNA1 and TWIST were also predicted to be important (Fig 5A). We linked the top 30 regulators to biological functions and found that tumorigenesis, cell movement and cell proliferation were the top processes, followed by cytoskeleton and cardiovascular disease (Fig 5B). It is perhaps unsurprising that these were the most prominent functional terms as both tumorigenesis and cushion formation share the common processes of EMT, cell migration and proliferation.

We then filtered our gene set for pathways associated with cardiac and development (using the terms card* and dev*), from the combined datasets from both time points (Figs 5 and S6A). Only the Regulation of the EMT in Development Pathway was significantly upregulated (more than three-fold compared to the myocardial and blood clusters) in the valve cluster. The data suggested NOTCH and WNT may be regulating the process of EMT, via SNAI2, CTTNB1, SNA1 and TWIST (Figs 5C and S6B). Developmental terms (trunk, genitourinary and vasculature) were all significantly positively associated with the valve cluster using the cardiac development filtering protocol. However, terms associated with “Congenital Anomaly of Cardiovascular System/Congenital Heart Disease” and “Familial Cardiovascular/Heart Disease” were significantly negatively associated (Fig 5D and 5E), although they were positively associated with the myocardial cluster.

### Novel pathways associated with arterial valve development

**Fig 6:**
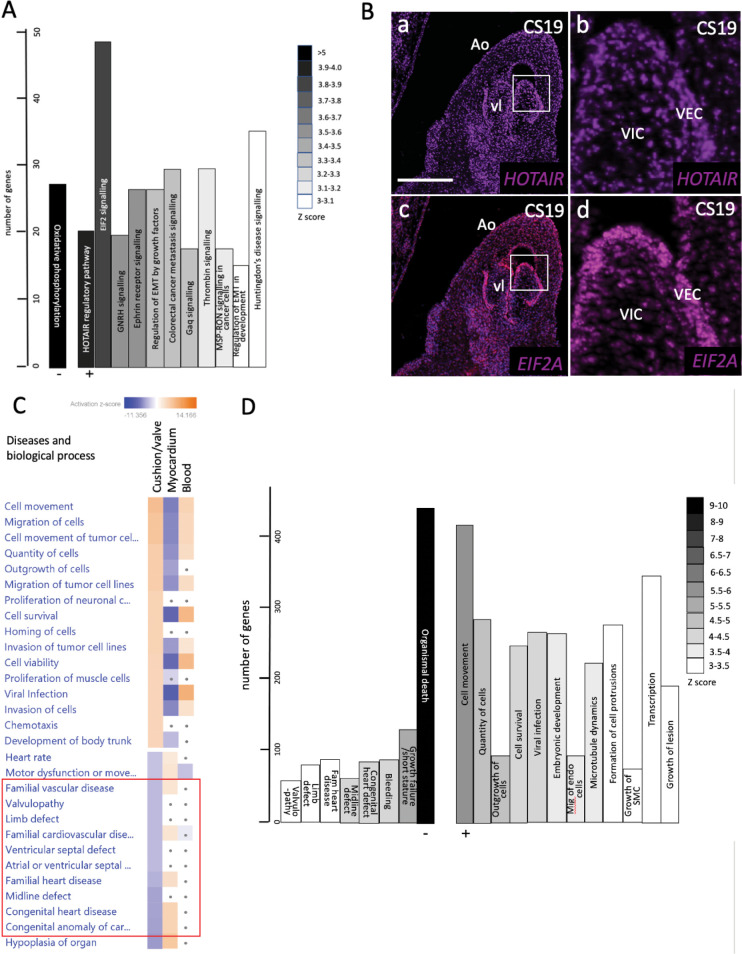
Functional associations of valve cluster genes generated by unfiltered IPA analysis. **A)** If no filtering is carried out, the HOTAIR and EIF2 signalling pathways are most strongly associated with the valve cluster data, whereas oxidative phosphorylation is negatively associated. **B)** Both HOTAIR and EIF2A are expressed in the valve endocardium (VEC) of developing aortic valves at CS19. HOTAIR is also expressed in VIC. The boxed area marks the area shown in high power (b,d). **C,D)** Cell movement/migration and proliferation-related terms are most strongly associated with the unfiltered valve dataset (compared with the myocardial), whereas terms relating to cell/tissue death and again congenital/familial heart/cardiovascular disease, including valvulopathy, (red box in C) were negatively associated. Scale bar in B = 200μm in a,c, 40μm in c,d.

In view of the single significant positively associated pathway (EMT), and the surprising negative association between the valve cluster genes and terms related to congenital heart disease, we carried out a second analysis, without filtering for cardiac and development, but otherwise using the same significance cut off of +/- two-fold compared to the myocardial and blood clusters. In this case, The HOTAIR Regulatory Pathway and EIF2 Signalling were the top two canonical pathways associated with the valve cluster (Figs 6A, and S7A and S7B). Although neither of these genes were found in our dataset, RNAScope analysis confirmed that HOTAIR and EIF2A were expressed in the endocardium of the developing human aortic valve (Fig 6B). GNRH Signalling, Ephrin Receptor Signalling and Regulation of Epithelial to Mesenchymal Transition were also significantly upregulated in arterial valve tissue. In the absence of filtering, the positively associated biological process and disease terms were involved in cell movement/migration and viability/survival/proliferation (Figs 6C and 6D and S7C). This fits well with the dynamic cell movements and rapid proliferation that is taking place in developing valve tissues at these developmental stages (reviewed in [25]). Negatively-associated terms were linked to growth failure or cell death, and similar to the filtered data, with familial heart disease, congenital heart defect and valvulopathy, although again, these were highly positively correlated in the myocardial cluster (Figs 6C and 6D and S7C). Notably, valvulopathy was also negatively correlated with our cushion/valve cluster.

### “Valve” genes are frequently associated with cardiac malformation and disease

Having identified a set of genes that were highly differentially expressed in human embryonic cardiac cushion tissue compared to the myocardium, but that were not well represented in the gene sets associated with congenital heart defects and cardiac development, we wanted to know if there was data in the literature to support an association between our gene list and heart malformation. We carried out a literature search using OMIM and PubMed to identify data linking the top 100 differentially expressed cushion genes and either human congenital heart disorders or cardiac defects in mouse knockouts (S5 Table). Breaking this data into quartiles, 15/25 genes in the top quartile were associated with congenital heart defects or diseases affecting the outflow region in mouse or human, compared with only 4/25 in the bottom quartile. We looked in more detail (using literature searches and interrogation of the Mouse Genome Informatics database; https://www.informatics.jax.org) at the phenotype of mouse knockouts of the novel genes LGALS1, PDLIM3, THBS1, LINC00632, RBP1, FLRT2, OLFML3, SLN, MXRA8, S100A11 and C7 that we highlighted earlier. Of these, there was no reported cardiac phenotype for LGALS1, OLFML3, MXRA8 and S100A11 mouse knockouts [39,40]. PDLIM3, THBS1 and FLRT2 knockout mice are reported to have cardiac defects although the valves were not specifically described [41–43]. SLN knockouts are reported to have abnormalities in cardiac contractility [44] but no congenital abnormalities were reported, whereas C7 mice are reported to have neurological disturbances (https://www.informatics.jax.org/allele/MGI:5823284). RBP1 knockout mice are reported to have cardiac metabolic disturbances but the anatomy of the heart does not appear to have been analysed in embryos or in adult animals [45,46]. Thus, there is a high incidence of great artery and valve malformations and/or disease associations with the most DEGs in our valve cluster, although in several cases null mice have not been investigated for these abnormalities.

### RBP1 knockout mice have abnormalities of the arterial valves

**Fig 7:**
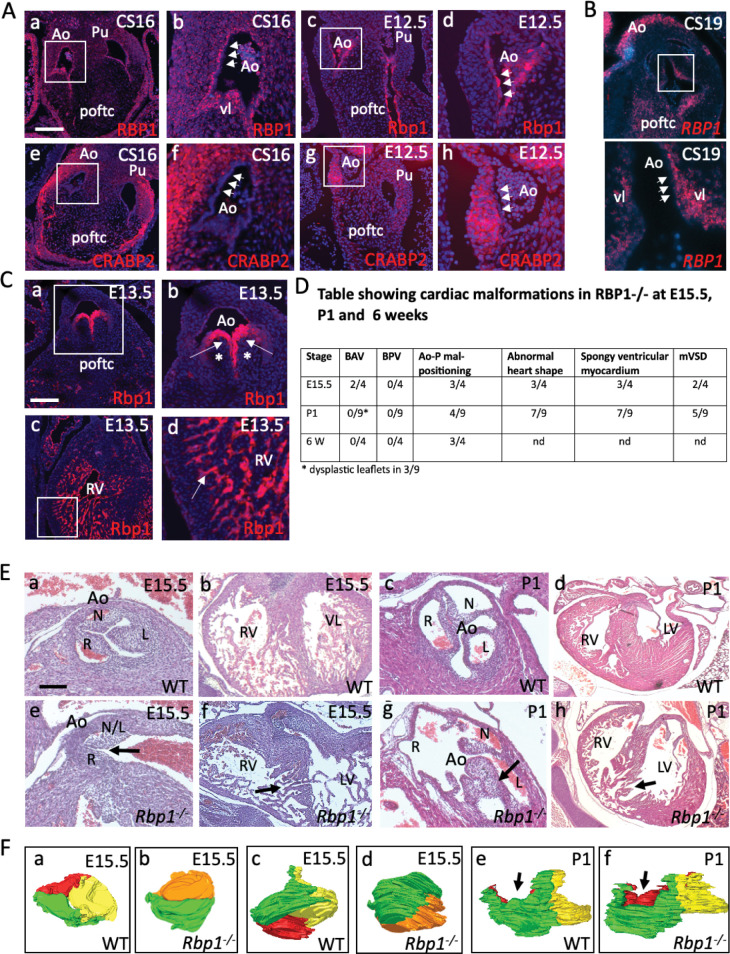
RBP1 plays a crucial role in the developing arterial valves. **A)** RBP1 and CRABP2 expression in the human and mouse heart. White boxes denote the area covered by the high-power images (b,d,f,h). RBP1 protein is expressed (red) in the cells underlying the endocardium (arrowheads) of the developing aortic and pulmonary valves in human CS16 hearts, as well as at lower levels in the vessel walls. In contrast, it is expressed in the endocardium (arrowheads) itself in the mouse heart and is not found in the vessel wall. CRABP2 expression (red) localises to the wall surrounding the valve leaflets in both species at the same timepoints but is not found in the endocardium (arrowheads). **B)** *RBP1* expression in a CS19 human embryo using RNAScope shows expression in the subendocardial region of the aortic valve leaflet (white arrowheads point to the endocardium). Expression can also be seen in the mesenchyme of the proximal outflow cushions and in the arterial wall. Boxed area shows the valve leaflets at higher magnification. **C)** Rbp1 protein is specifically localised to the endocardium of the developing valve leaflets and cardiac chambers at E13.5 in the mouse heart. It is not found in the interstitium of the developing valve leaflets (*). **D)** Table showing cardiac malformations found in *Rbp1* knockout animals. **E)** Abnormalities of the aortic valve including BAV (in the E15.5 mutant; e) and valve dysplasia (arrow in P1 mutant; g) were seen in late fetal and neonatal RBP1 mutants. *Rbp1* mutant neonates also have abnormalities on the ventricular myocardium including muscular ventricular septal defects (arrows in f,h). Stage-matched and oriented control aortic valves are shown for comparison. **F)** 3D reconstructions of the aortic valve of wild type and *Rbp1* null mutants at E15.5 (a-d) and P1 (e,f). WT and *Rbp1-/-* mutants are matched for orientation. Red = non-coronary leaflet, yellow = left leaflet, green = right leaflet. Orange is a fused non-coronary and left leaflet. a-d) Three leaflets were seen in the aortic valve of WT at E15.5 (a,c) compared to two leaflets observed in a *Rbp1-/-* observed from above (a,b) and from the right side (c,d). e,f) three leaflets are seen in both the WT and *Rbp1-/-* at P1, although abnormalities in the shape and position of the leaflets means that the leaflets are not all at the same level (arrows in e,f). Ao = aortic valve; L = left leaflet; L/N = left-non-coronary fused leaflet; LV = left ventricle; N = non-coronary leaflet; poftc = proximal outflow tract cushions; Pu = pulmonary valve; R = right leaflet, RV = right ventricle. Scale bar in A = 100μm in a,c,e,g, 35 μm in b,d,f,h; 150μm in B; in C = 75μm in a, 40μm in b, 50μm in c, 20μm in d; in E = 50μm in a,c,e,g, 150μm in b,f, 300μm in d,h.

We were intrigued that RBP1 and CRABP2 (cellular retinoic acid binding protein 2) were both found in our list of the top 30 most DEGs in the valve cluster (Fig 3A) and that RARβ was one of the top 30 upstream regulators (Fig 5A) of the valve cluster. Although the retinoic acid signalling pathway has not previously been specifically associated with arterial valve development, we first searched GenePaint to establish whether there was evidence that retinoid signalling might be important in arterial valve development. This revealed a number of key genes, including RALDH3, RARA, RARB, RARG, RXRB and CRABP1, as well as a number of important enzymes and downstream targets, are expressed in the arterial valves (S8 Fig). With this confirmation, we examined the expression patterns of RBP1 (also known as CRBP1) and CRABP2 in more detail in both mouse and human embryos Fig 7A and 7B). RBP1 protein was specifically localised to the subendocardial region of the arterial valves in human at CS16-19, although it was expressed in the valve endocardial cells at E11.5-E12.5 in the mouse. There was also RBP1 protein expression found in the walls of the arterial roots, the atrial myocardium and to a lesser extent in the ventricles in the human embryo. RNAscope analysis in CS19 human embryos showed a similar pattern, with expression in the subendocardial region of the valve leaflets, the mesenchyme of the proximal outflow tract and in the arterial wall. In contrast, CRABP2 was found in the walls of the arterial roots in both species at CS16-19 in human / E11.5-E12.5 in mouse, with only minimal expression in the endocardium (Fig 7A).

Whereas the mouse model for CRABP2 has been described in detail, with only mild digit defects reported [47], the embryonic phenotype of *Rbp1* knockout mice does not appear to have been analysed in any detail [45,46]. We therefore looked at *Rbp1* expression in the mouse at E13.5, showing specific expression in the valve endocardium, and in the endocardium of the atria and ventricles. We obtained *Rbp1* knockout fetuses and neonates at E15.5 and P1 respectively and analysed the hearts for structural heart defects. Analysis of *Rbp1* knockout fetuses at E15.5, comparing them to wild type animals on the same genetic background, revealed a range of defects affecting the ventricular myocardium and the outflow region of the heart (Figs 7D and 7F and S9). These included abnormalities in the positioning of the great arteries so that the arteries were parallel in 2/4 mutants analysed, and a tortuous pulmonary trunk in 3/4. Moreover, BAV (see three-dimensional reconstructions of the valve leaflets; Fig 7F) was found in 2/4 RBP1 knockouts examined at E15.5 (Fig 7E and 7F)). Nine *Rbp1* knockouts were also examined at P1 (Fig 7D and 7F), with dysplastic aortic valve leaflets seen in 3/9 Fig 7E and 7F). Mal-alignment of the aorta and pulmonary trunk (including parallel arterial trunks) was observed in 4/9 of the P1 *Rbp1* mutants examined (Fig 7D) and was also observed in 3/4 *Rbp1* mutants examined at 6 weeks of age (Fig 7D). Ventricular septal defects affecting the muscular part of the septum were also a common finding, affecting 7/13 of the E15.5 and P1 mutants examined. No malformations were seen in wild type animals (E15.5 n = 4; P1 n = 9; 6 weeks n = 4). We looked at the breeding records of the *Rbp1* knockout mice. Whilst it had not been noted that there was any neonatal loss, in crosses of *Rbp1* knockout males and females (97 litters), the average litter size was 5.05+/-2.56, whereas in a backcross of the *Rbp1* knockout to C57Bl/6N (giving heterozygous pups; 18 litters), the litter size was 6.59 +/-2.85. Thus, it is possible that some of the *Rbp1* knockout mice die in the immediate neonatal period (and are cannibalised by the mother), potentially of cardiac malformations. Surviving *Rbp1* knockout mice appeared healthy although the mice were only maintained up to the age of 1 year.

## Discussion

ST is becoming more popular as a technique for gathering spatially-related transcriptomic data [28,31,32]. The potential to measure transcript expression levels whilst maintaining the spatial context from which they arose, together with the new insights into cellular organisation this data reveals, is an obvious biological research goal with many large-scale consortia (e.g. Human cell Atlas, Human Developmental Biology Initiative) utilising ST as a key technology to realise this aim. However, ST, as a method, is still in its infancy. As such, no clear recommendations on best practice have yet emerged. One key aspect of the method that has yet to be resolved is the number of replicates required to produce robust datasets. Although we did not see major differences between our technical and biological replicates it is important to remember that, as with any transcriptomic study, the number of replicates influences the statistical power of the experiment [48]. Biological replicates are needed to detect DEGs between conditions, whereas technical replicates provide a means to distinguish true differences in gene expression from experimental artefacts. We have taken our lead from that of single cell RNA sequencing and have adopted an approach of using at least three technical replicates (tissue sections) for each tissue sample. This strategy acts to increase the number of spots for each given spatial position and therefore increases the transcriptional data obtained at that position, thereby statistically powering up the assay. In an ideal world, a similar approach would be taken for the biological replicates, with a suggestion that a minimum of three samples at each specific sampling time point should be used. However, it is acknowledged that obtaining rare patient samples, or as in this study, early human embryos, is often challenging. Even with the increased resolution of new ST technologies (e.g. 10X Genomics Visium, see below), technical replicates will remain an important consideration, particularly for studies where the area of interest is small or small cell populations fall between the spot capture areas, as we saw with our analysis of the valve leaflets. It will be interesting to see how the community responds to address the question of the number of replicates required for confident analysis of the data as more researchers adopt ST.

The technologies for performing ST are continuing to be developed and improved upon and there are now multiple commercial ST applications available. Indeed, the method used here has now been superseded by release of the 10X Genomics Visium kits which have doubled the spot resolution. Regardless, the data presented here is still very much of value, especially given the limited number of other outflow cushion-specific human datasets and given the validation by *in situ* methods and overlap with existing human and mouse data. Despite the lower resolution of our study, we found that our use of technical replicates provided us with enough datapoints to distinguish three spatially distinct biological regions. However, even with these replicates we were unable to study individual valve leaflets in detail. Statistically significant data between cushion-derived and intercalated valve swelling-derived valve leaflet precursors was not achieved and points towards higher resolution studies being required to address this when the technology is capable of single cell resolution in the future. Such advancements in ST are already underway and the spatial resolution of the technology can, or very soon will, be able to resolve to a scale smaller than that of a single cell (for review see [49]). However, this does not on its own mean that single cell resolution can be achieved for ST from tissue sections. It is extremely difficult to section any tissue uniformly to a single cell thickness and therefore the data sampling points are always likely to contain transcriptomes from more than one cell. The sequencing costs involved in the scale up to one cell resolution are also likely to be prohibitive. It is perhaps expected therefore, that the move to single cell resolution for ST will come from a combination of increased resolution of the ST assay in conjunction with advances in computational integration of scRNA-seq and ST data—the single cell resolution coming from the data obtained from sc-RNA seq and the spatial positioning from ST. Strides have already been made in this regard with software packages such as Cell2location, MERINGUE and SquidPY [50–52], already allowing for such combined analysis; new applications enabling more sophisticated analysis are anticipated.

Comparison between the transcriptomes of the combined clusters from CS16 and CS19 hearts showed that the two datasets showed a high degree of similarity, although separated by approximately 8–10 days. A related study using human embryos [31] came to a similar conclusion and merged data from 4-5-9 post conceptional weeks. Although the core genes expressed were very similar at CS16 and CS19, there were on average more than 50% more genes captured at CS19 compared to CS16. When CS16 and CS19 valve cluster gene lists were compared, there was less overlap (approximately 25% of genes) than expected based on the whole dataset (three clusters combined). However, the shared CS16/CS19 genes showed higher specificity for the valve region than did the stage-specific genes (see S4 Fig), suggesting the combined dataset is more robust. Between these Carnegie stages the outflow tract is actively growing and undergoing septation, although these processes are not complete for another 3–5 days of gestation beyond CS19. Thus, although heart morphology changes over this time frame, the genes regulating and supporting this morphogenesis overlap to a high degree. Three non-overlapping (in terms of spots from CS16 and 19 hearts) clusters were identified at these time points. Analysis of the most DEGs, as well as “perfect marker” analysis, confirmed these as myocardial, valve and blood (in the lumen of the heart). Notably, we could not establish a distinct endocardial dataset, with genes for this cell type found in all 3 clusters. This likely reflects the large relative size (100μm) of the spots, which is larger than the typical thickness (10–30μm) of the endocardial monolayer. More recent versions of the technique have significantly better resolution (55μm) although it is likely that resolution of the endocardial monolayer will continue to be a problem until single cell resolution is achieved. Strikingly, 29/30 of the top DEGs in the myocardial cluster, and 28/30 of the genes in the blood cluster, were already known to be abundantly expressed in these tissues. In contrast, 11/30 of the DEGs in the valve cluster had not previously been linked to the cardiac valves, at least until transcriptomic studies came along (see Fig 3), suggesting that this tissue is less well annotated at the transcriptional level than are myocardium or blood. This would appear to be supported by the fact that IPA analysis showed that the genes in the valve cluster were negatively associated with terms linked to congenital heart disease and familial heart disease anomalies (Fig 5). In contrast, our myocardial cluster was strongly correlated with these terms. This surprising result suggests that datasets linked to congenital heart disease/familial heart disease over-represent genes expressed in cardiomyocytes (which are the major cell type in the developing heart), compared to those expressed in valve tissue. For large genomic studies, some degree of filtering is essential in order to whittle down the huge number of variants detected to a workable number for further analysis. The unintended outcome from this is that mostly cardiomyocyte genes will be positively selected when BAV/CHD genomic data are filtered for “cardiac development” terms, because valve-related genes are under-represented in the relevant datasets. As a consequence, potential causative variants may be excluded from further analysis. Without wanting to over interpret these results, this may explain why relatively few causative genes have been identified by genomic studies of CHD cases, and even fewer have been validated as being disease causing.

Extracellular matrix molecules, cytoskeletal proteins and transcriptional regulators were the commonest classes of protein encoded by the most abundant genes in the valve cluster, participating in cellular processes such as EMT (EndMT in the cushions/valves), cell movement/migration and proliferation–all processes that are known to be crucial in the remodelling valves (reviewed in [19,53]) and other reviews). Importantly, our data overlaps and complements the existing datasets for developing valves generated from mouse tissue from a range of embryonic and postnatal stages. For example, genes associated with bone formation were highly represented in our valve dataset, as has been reported for mid-late fetal mouse valves [33]. Similarly, considerable overlap was found with our dataset and single cell RNASeq data from postnatal mouse valve interstitial cells [36]. Comparisons with the first trimester human outflow tract and atrioventricular valves [31], revealed 25–30% overlap between the two datasets for the top 250 genes in each. Thus, our data is well supported by data generated from other studies and adds an important transcriptional dataset representing the developing human arterial valves.

Variants in several of the most DEGs in the valve cluster, including COL1A2, ELN and FBLN1 [54–56], have been suggested as being potentially causal for BAV in human genomic studies, whereas MFAP4 and GPC3 have been implicated as causal genes for other CHD [57,58]. Notably though, COL1A2, ELN and FBLN1 are all expressed in the arterial wall surrounding the valve (Fig 3) and are primarily implicated as causal genes for aortopathy, with their roles as causal genes for BAV unproven. Although several of the highly DEGs we identify have not previously been shown to be expressed in the developing valve region, almost all, where expression data was available, were confirmed as being expressed in the arterial roots where the valves are located. Thus, the ST appears to have successfully identified the arterial valve transcriptome at the CS16-19 stage of human development. Four genes (STX10, HES4, MXRA5 and LINC00632) in the top 250 DEGs in the arterial valve cluster are not found in the mouse genome and therefore could not have been detected from mouse-based transcriptomic studies. The expression patterns of these genes all confirmed expression in the arterial roots, with MRXA5 and LINC00632 found in the forming valve leaflets. HES4 is an important downstream target of Notch signalling and has been identified as being differentially expressed in VIC from calcific aortic valve disease [59]. MRXA5 has been reported to be expressed in cushion tissue in the chicken embryo [60] although nothing (until now) was known about its expression pattern in developing human heart. All of these genes can now be considered potential candidates for human arterial and valve disease.

IPA analysis, whether filtering the data for cardiac and development, or without this filter, highlighted CXCL12, TGFBR1 and more generally, pathways involved in regulating EMT, in the remodelling phases of valve development. As already mentioned, EndMT is a key process in cushion/valve development and this process is initiated by the TGFβ signalling pathway (reviewed in [53]). Although EndMT should be mostly completed by CS16 (equivalent of mouse E12.5), most of these genes continue to play roles during the remodelling stages of valve development (reviewed in [19,53]). CXCL12 has also been shown to play an early role in the directional migration that is a crucial part of EndMT, but also in regulating cell proliferation at later remodelling stages [37]. Similarly, TGFBR1 (ALK5) also plays crucial roles in valve development [61]. The unfiltered analysis also suggested that HOTAIR and EIF2 signalling may play important roles in valve development, although there is nothing known about these pathways in the developing heart. We were particularly intrigued by the HOTAIR pathway as this is a long non-coding RNA that regulates HOX gene expression [62]. HOXA3 was highly differentially expressed in the valve cluster, appearing in the top 30 transcriptional regulators (S6B Fig). Interestingly, null mutants for HOXA3 (then called hox1.5) are reported to have cardiac defects including BAV [63], confirming that this gene is vital for arterial valve development. HOX genes are generally thought to be regulated by retinoic acid signalling in the developing heart [64,65], so we were interested that two genes in this pathway, RBP1 and CRABP2, were amongst the top 30 DEGs in the valve cluster, with immunohistochemistry confirming their localisation to the developing arterial valve leaflets in the human and mouse. Moreover, RARβ was predicted to be one of the most important regulators of genes in the cluster. Although there is considerable evidence that retinoic acid plays an important role in endocardial cushion development, its precise roles remain unclear. Excess and deficient RA signalling disrupts formation of the endocardial cushions [66,67], at least in part through the dysregulation of key genes, such as Tbx2 [68]. RA has also been shown to be critical for the development of the coronary vasculature [69]. Given these findings, it seems that RA levels need to be tightly controlled for normal development to proceed. Analysis of RBP1 knockout fetuses, neonates and adults revealed a range of defects affecting the outflow of the heart, particularly the arterial valve leaflets, the alignment of the aorta and pulmonary trunk and the ventricular myocardium. Thus, RBP1, and presumably RA signalling more generally, appears to be crucial for development of the outflow tract including the arterial valve leaflets and thus are potential causative genes for BAV and other valve malformations.

This study has shown the utility of ST for generating arterial valve-specific transcriptomic data from human embryos, broadening our knowledge of the genes and signalling pathways important in human valve development, and acting as a foundation for more targeted screening of variants for common arterial valve malformations such as BAV.

## Materials and methods

### Ethics statement

Human embryos were obtained from the Human Developmental Biology Resource (HDBR) following social terminations of pregnancy with informed donor consent (written consent was obtained in each case) and ethical approval from the Newcastle and North Tyneside 1 National Health Service (NHS) Health Authority Joint Ethics Committee (08/H0906/21+5).

### Human and mouse embryos

Human heart samples, Carnegie stage (CS)16 and CS19, were dissected from the embryo, embedded into Optimal Cutting Temperature (OCT) and snap-frozen in an isopentane bath on liquid nitrogen. Sections were taken using a Leica cryostat at 10μm intervals, RNA extracted from four consecutive tissue sections and RNA Integrity Number (RIN) analysis performed using an Agilent Bioanalyzer. Samples with a RIN value of 7 or above were deemed suitable for ST analysis.

Mouse embryos (CD1 or C57Bl/6) were obtained from timed matings carried out overnight, with the presence of a copulation plug designated embryonic day (E) 0.5. *Rbp1*^–/–^mice (C57BL/6N background) were bred similarly according to institutional guidelines of the University of Maryland, Baltimore. *Rbp1*^–/–^mice were originally obtained from Pierre Chambon and Norbert Ghyselinck (Institut de Genetique et de Biologie Moleculaire et Cellulaire, Institut National de la Santé et de la Recherche Medicale, Illkirch, France). Mice were maintained according to the Animals (Scientific Procedures) Act 1986, United Kingdom, under project license P9E095FF4. All experiments were approved by the Newcastle University Ethical Review Panel.

### Spatial transcriptomics

An overview of the ST methodology and workflow employed is shown in Fig 1C. 10μm tissue sections were collected into the capture windows of an ST Tissue Optimisation slide (10X Genomics cat. no. 1000131) and the permeabilisation conditions for the ST analysis of the heart tissue determined following the method described in the Tissue Optimisation Manual (Spatial Transcriptomics, version 190219). Briefly, this involved pre-permeabilisation of the sections in 50U/μl collagenase at 37°C for 20 minutes, followed by permeabilisation using a staggered sequence of 0–12 minute intervals in 0.1% pepsin/HCl at 37°C. RNA captured by the oligo-dT probes bound to the slide was reverse transcribed and Cy3-labelled nucleotides incorporated into the reaction. The optimal permeabilisation time was then determined by visual inspection of the resultant fluorescent cDNA footprint on each section.

Consecutive (serial) tissue sections in the regions containing the arterial valves (5 at each time point) were then sectioned to an ST Library Preparation slide (10X Genomics, cat # 1000132), H&E stained (S1 Fig), and the tissue sections and underlying spots (each spot covering a 100μm region and separated by distance of 200μm from the centre of each neighbouring spot) captured using a Nikon TiE inverted microscope at 10x magnification (Plan Fluor 10x 0.3 NA). Each spot, based on our measurements of CS16 cushion mesenchyme/valve interstitial cells, covered approximately 50 cells. Raw images were stitched together using NIS-Elements software with parameters set to 20% overlap and stitching via optimal path method. ST analysis was performed as per the manufacturer’s instructions (Spatial Transcriptomics Library Preparation Manual, version 190219). Following enzymatic cleavage of the barcoded probes and associated captured RNA transcripts from the slide, sequencing libraries were generated and sequenced at 50,000 reads per sampling region (spot) using an Illumina NovaSeq 6000.

### Alignment and quantification

The paired FASTQ files were de-multiplexed with a publicly available perl script (https://github.com/tallulandrews/scRNASeqPipeline/blob/master/0_custom_undo_demultiplexing.pl) using the spatial barcodes encoded in read 1. Read 2 from successfully de-multiplexed pairs were trimmed for quality using Trimmomatic version 0.3 [70]. A reference was created using Ensembl human reference genome (GRCh38.p12). The trimmed reads were aligned to the reference using STAR version 2.5.3a in single read alignment mode [71]. The number of reads were quantified using HTSEQ version 0.6.1 [72] and a count matrix was created. Spot detector software [73] was used to align the fluorescent image with the brightfield image to determine the pixel co-ordinates for the spots.

### Quality control and visualisation using spaniel R package

We developed the Spaniel R package [74] which provides a framework for quality control, visualisation and pre-processing of ST data. Spaniel is available from bioconductor (https://www.bioconductor.org/packages/release/bioc/html/Spaniel.html) and is built on existing S4 objects namely the SingleCellExperiment object and Seurat object to facilitate the use of other single cell methodologies. The gene expression matrix was imported in R using the Spaniel package and quality control steps were performed to remove spots which fell outside the tissue area. We assessed the number of genes per spot, number of reads per spot and percentage of mitochondrial reads per spot. High levels of mitochondrial genes can indicate poor sample quality leading to abnormal gene expression patterns caused by the capture process rather than underlying biology of the sample itself. Samples with low quality are also likely to have low numbers of gene or reads detected. Section 2 of the CS16 tissue was removed from analysis because of quality control issues, leaving 4 sections for analysis at CS16 and 5 at CS19.

### Clustering analysis and visualisation of gene expression

Gene expression was normalised and highly variable genes were identified using Seurat. We firstly combined the technical replicates from the adjacent tissue sections into one dataset using Seurat integration methods to account for batch effects from the data [75]. Secondly, the spots from all sections at each time point were combined into one dataset, again using Seurat integration methods. Clustering analysis was performed on each of the integrated datasets with a resolution of 0.8. Differential Expression analysis was performed to identify changes in gene expression between the clusters using the Wilcoxon test.

### IPA analysis

Pathway analysis was performed using Qiagen Ingenuity Pathway Analysis (IPA) software. The marker gene lists for each of the three clusters, with an adjusted p value of less than 0.05 were run through the core analysis module of IPA. A comparison analysis was then performed to look for differences and similarities between the canonical pathways, upstream regulators and disease terms associated with each set of marker genes. A significance threshold of an absolute value of 2 for the z score was set where a z-score of 2 or higher indicates that a pathway is activated and –2 or lower indicates that is inhibited. The pathways were filtered for just those containing the terms cardiac or development (using the wildcard *card* or *dev *). Network plots were generated using IPA software.

### Fluorescent immunohistochemistry

Methodology was as published previously [15]. Sections were cut from paraffin-embedded mouse and human embryos (different to those used for the ST analysis) at 8 μm using a rotary microtome (Leica). Slides were de-waxed with Histoclear (National Diagnostics) and rehydrated through a series of ethanol washes. Following washes in PBS, antigen retrieval was performed by boiling slides in citrate buffer (0.01 mol/L) pH 6.3 for 5 minutes. Sections were blocked in 10% FCS and then incubated overnight at 4°C with the following primary antibodies diluted in 2% FCS: LGALS1 (Abcam ab138513), RBP1 (Invitrogen PA528713), CRABP2 (Abcam ab211927). After washing, sections were incubated at room temperature for one hour, with secondary antibodies conjugated to Alexa 568 (Life Technologies). Fluorescent slides were then mounted with Vectashield Mounting medium with DAPI (Vector Labs). Immunofluorescence images were collected with using a Zeiss Axioimager Z1 fluorescence microscope equipped with Zeiss Apotome 2 (Zeiss, Germany). Acquired images were processed with AxioVision Rel 4.9 software.

### RNAScope

All reagents used were taken from the RNAscope Multiplex Fluorescent Reagent Kit v2 (bio-techne, cat# 323100). 8μm sections were cut from CS16 and CS19 formalin-fixed paraffin-embedded embryos (different to those used for the ST analysis) using a Leica microtome. The slides were heated to 60°C for 10 min and allowed to cool to room temperature before dewaxing in xylene, rinsing in two changes of 100% ethanol and air drying. Following incubation in hydrogen peroxide at room temperature for 10 minutes and rinsing successive changes of water, the slides were then submerged in Target Retrieval solution and placed in a boiling water bath for 20 minutes. After rinsing the slides in 100% ethanol and air drying, protease plus solution was added to the sections for 30 minutes at 40°C. RNAscope probes were diluted into either probe diluent (bio-techne, cat # 300041) or a channel 1 probe as appropriate in a 1 in 50 volume, and the slides incubated at 40°C for 2 hours. Following sequential amplification steps with AMP1 and AMP2 solutions, the slides were incubated with HRP-C1 at 40°C for 15 minutes, rinsed in wash buffer and incubated at 40°C for 30 minutes in OPAL flurophore 520 (Akoya Biosciences) diluted 1 in 100 with TSA diluent. After blocking for 15 minutes at 40°C with HRP blocker, the same steps were repeated with HRP-C2 solution and OPAL flurophore 570 (Akoya Biosciences). After nuclei staining with DAPI, slides were mounted in Prolong Gold Antifade (Thermo Fisher Scientific) and imaged as above. Human RNAscope probes used in this study (bio-techne): PDLIM3 (cat. # 533411-C3), ID4 (cat. # 466371), STX10 (cat. # 1119651), HES4 (cat. # 548621-C3), MRXA5 (cat. # 419691-C2), HOTAIR (cat. # 312341-C2), EIF2A (cat. # 410701), RBP1 (cat. # 484751).

### BaseScope

8 μm sections were cut from formalin-fixed-embedded embryos using a Leica microtome. Slides were heated to 60°C and allowed to cool to room temperature. Slides were then dewaxed in two changes of xylene (5 minutes each), rinsed in two changes of 100% ethanol followed by air drying. Sections were covered with hydrogen peroxide and incubated at room temperature for 10 minutes and rinsed in two changes of diethyl pyrocarbonate (DEPC) treated water. Following incubation and rinsing, slides were then placed in boiling target retrieval solution for 20 minutes, then immediately rinsed in two changes of DEPC water. After rinsing, slides were rinsed in two changes of 100% ethanol and allowed to air dry. Protease IV was added to cover sections, and incubated for 30 minutes at 40°C. Following rinsing in 2 changes of DEPC water, two drops of channel 1 BaseScope probe BA-Hs-LINC00632-213-2zz-st-C1 (bio-techne, cat# 1128991-C1) was added to sections and incubated at 40°C for 2 hours. Following 8 sequential amplification steps with AMP1 –AMP8, 1:60 BaseScope Fast RED-B was added to BaseScope Fast RED-A and pipetted onto sections (BaseScope v2 Red Assay—bio-techne, cat# 323910). After a 10 minute incubation period, slides were rinsed in DEPC water and counterstained with 50% methyl green solution (Vector, Cat# H-3402). Slides were then heated to 60°C for 30 minutes, mounted with EcoMount (Vector, cat# H-5000), coverslip and imaged as above.

## Supporting information

S1 Fig H&E staining of human valve sections used for ST analysis. A) Isolated hearts were sectioned in the frontal plane. Sections are sequential. B) Enlarged sections showing tissues present in sections. oftc = outflow tract cushions, oftw = outflow tract walls, rbc = red blood cells (in lumen), v = venticles, vlp = valve leaflet primordia.
(TIF)

S2 Fig Mapping back of perfect markers to CS19 human valve sections. Perfect marker genes (see Fig 2) for a range of cardiac progenitor cell types (neural crest cells), differentiated cardiac cell types (cardiomyocytes, cushion tissue, fibrous tissue, endocardium, smooth muscle cells), haematopoietic cell types that are known to be found in the heart (red blood cells, lymphocytes, macrophages) and those that are known to be similar to cushion tissue (bone, cartilage), were mapped back to the CS19 human valve sections used for the ST analysis.
(TIF)

S3 Fig **A) GenePaint images for all of the top 30 cushion cluster genes (as shown in Fig 3A) and B) RNAscope images of ID4 and PDLIM3 at CS19.** A) The whole embryo for each gene is shown on the left and a higher magnification image of the heart is shown on the right. Blue arrows in the Col 1A2 images point to the arterial valves whereas the red arrows point to the atrioventricular valves. Similar images are shown for each gene. For almost every gene (with the exception of C7), high level expression is seen in the valve tissue. B) Arrows point to regions of high-level expression in the pulmonary valve.
(EPS)

S4 Fig Top 30 valve genes in the CS16 and CS19 datasets. A) Top 30 DEGs in the CS16 (left list) and CS19 (right list) compared to the combined dataset. B) At both stages, GenePaint data suggested that genes already known to be expressed in the valve region (blue letters) were clearly and specifically expressed in the valve region, whereas novel genes were less strongly expressed (black letters) or were undetectable, particularly at CS16 (e.g. CS16: RAB32, TIPARP, ZBT58A; CS19:GLT8D2).
(TIF)

S5 Fig PANTHER designation of Top250 genes in valve cluster based on A) protein class and B) biological process. Pie charts showing A) the most common gene/protein classes and B) associated biological processes for the genes in the combined CS16/CS19 valve dataset determined using PANTHER. Gene Ontology (GO) codes are in brackets.
(TIF)

S6 Fig **IPA analysis on the “cardiac development” filtered CS16/CS19 combined dataset showing all statistically significant A) canonical pathways and B) upstream transcriptional regulators.** A) canonical pathways and B) upstream transcriptional regulators identified using Ingenuity Pathway Analysis (IPA) to scrutinise the CS16/19 combined dataset. Filtering for “cardiac” and “development”. This complements the data provided in Fig 5.
(TIF)

S7 Fig **IPA analysis on the unfiltered CS16/CS19 combined dataset showing all statistically significant A) canonical pathways, B) upstream transcriptional regulators and c) diseases and biological processes.** A) canonical pathways, B) upstream transcriptional regulators and c) diseases and biological processes identified using Ingenuity Pathway Analysis (IPA) to scrutinise the CS16/19 combined dataset. No filtering was applied. This complements the data provided in Fig 6.
(TIF)

S8 Fig GenePaint images for genes within the retinoid pathway, expressed in the arterial valves. The whole embryo for each gene is shown on the left and a higher magnification image of the heart is shown on the right. In each case, the arrows point to the arterial valve.
(TIF)

S9 Fig 3D reconstructions of WT and *Rbp1-/-* aortic valves in position within the aortic root. 3D reconstructions of the aortic valve of wild type and *Rbp1* null mutants at E15.5 and P1 placed within the aortic root (grey). WT and *Rbp1-/-* mutants are matched for orientation. Red = non-coronary leaflet, yellow = left leaflet, green = right leaflet. Orange is a fused non-coronary and left leaflet. At E15.5, three leaflets were seen in the aortic valve of WT at E15.5. In comparison, two leaflets observed in a *Rbp1-/-* mutant (Mut 1) observed from the right side. In the other mutant (Mut 2) shown, three leaflets were observed although two were fused along the majority of their length. In this latter case, it is clear that the proximal extent of the leaflets is not the same (arrows). Three leaflets are seen in both the WT and *Rbp1-/-* at P1, although abnormalities in the shape and position of the leaflets are apparent.
(TIF)

S1 Table DEGs in the combined CS16/19 myocardial cluster. Genes are ordered from highest (most differentially expressed) to lowest.
(XLSX)

S2 Table DEGs in the combined CS16/19 cushion/valve cluster. Genes are ordered from highest (most differentially expressed) to lowest.
(XLSX)

S3 Table DEGs in the combined CS16/19 blood cluster. Genes are ordered from highest (most differentially expressed) to lowest.
(XLSX)

S4 Table Comparisons between the valve cluster and published datasets. Sheet 1: comparison of top 250 DEGs from Queen data (this manuscript) to DeLaughter et al (2013). Shared genes are in red. Sheet 2: list of top 250 DEGs from Queen data (this manuscript) and Asp et al (2019) clusters 5 and 6. Sheet 3: comparison of top 30 DEGs from Queen data (this manuscript) to Asp et al (2019) clusters 5 and 6. Shared genes are in green. Sheet 4. comparison of top 250 DEGs from Queen data (this manuscript) to Asp et al (2019) clusters 5 and 6. Shared genes are in green.
(XLSX)

S5 Table Top 100 DEGs in the valve cluster linked to cardiovascular phenotypes in human patients and in mouse mutants. Dark yellow suggests an arterial valve phenotype, lighter yellow reflects a cardiovascular phenotype.
(XLSX)
