# Supplementary figures and images for: Spatial transcriptomics reveals novel genes during the remodelling of the embryonic human arterial valves
Source: PLoS Genet. 2023 Nov 27;19(11):e1010777. doi: 10.1371/journal.pgen.1010777 (PMC10703419; doi:10.1371/journal.pgen.1010777)

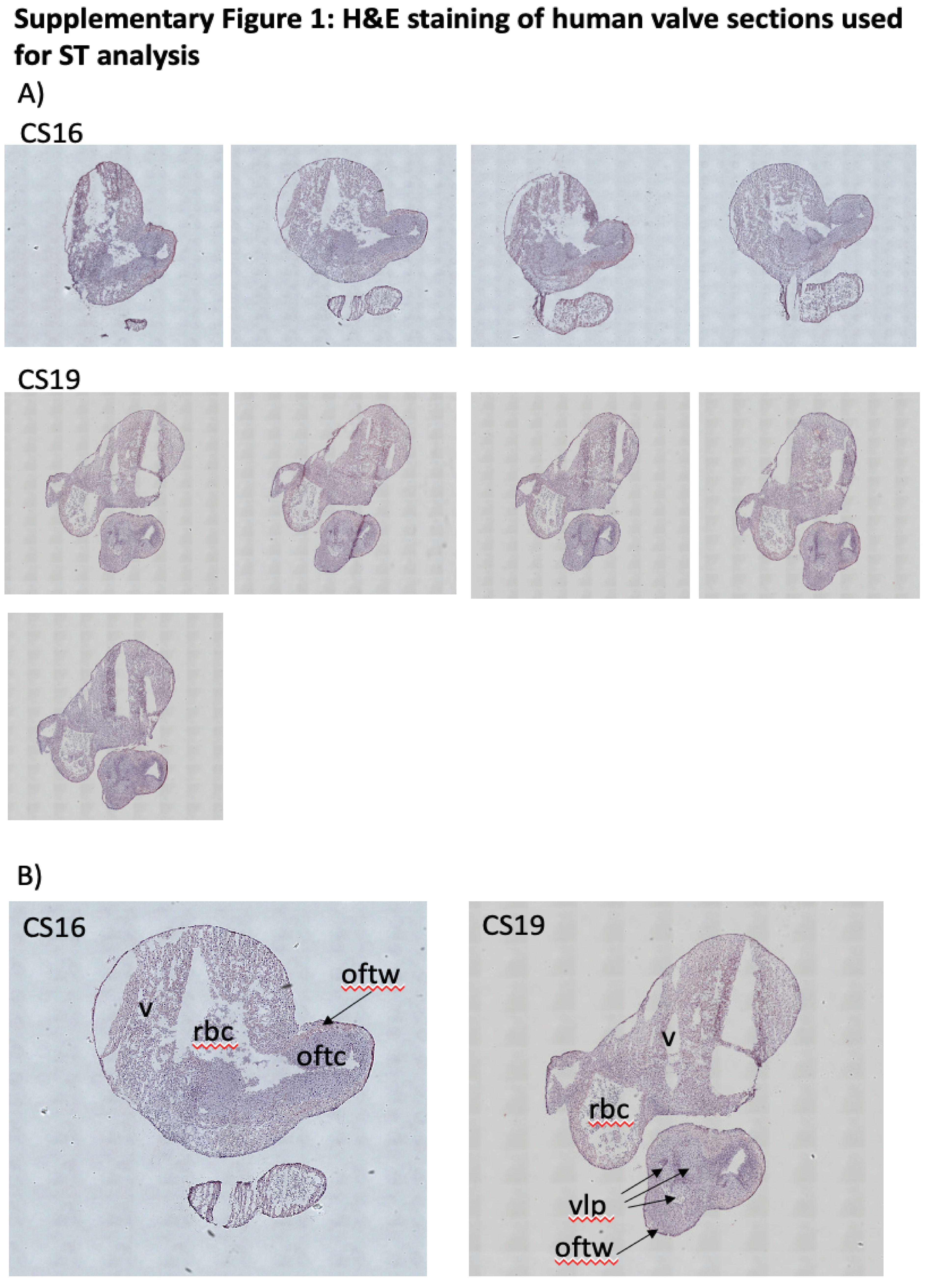

Supplement: S1 Fig — A) Isolated hearts were sectioned in the frontal plane. Sections are sequential. B) Enlarged sections showing tissues present in sections. oftc = outflow tract cushions, oftw = outflow tract walls, rbc = red blood cells (in lumen), v = venticles, vlp = valve leaflet primordia. (TIF) [file pgen.1010777.s001.tif]

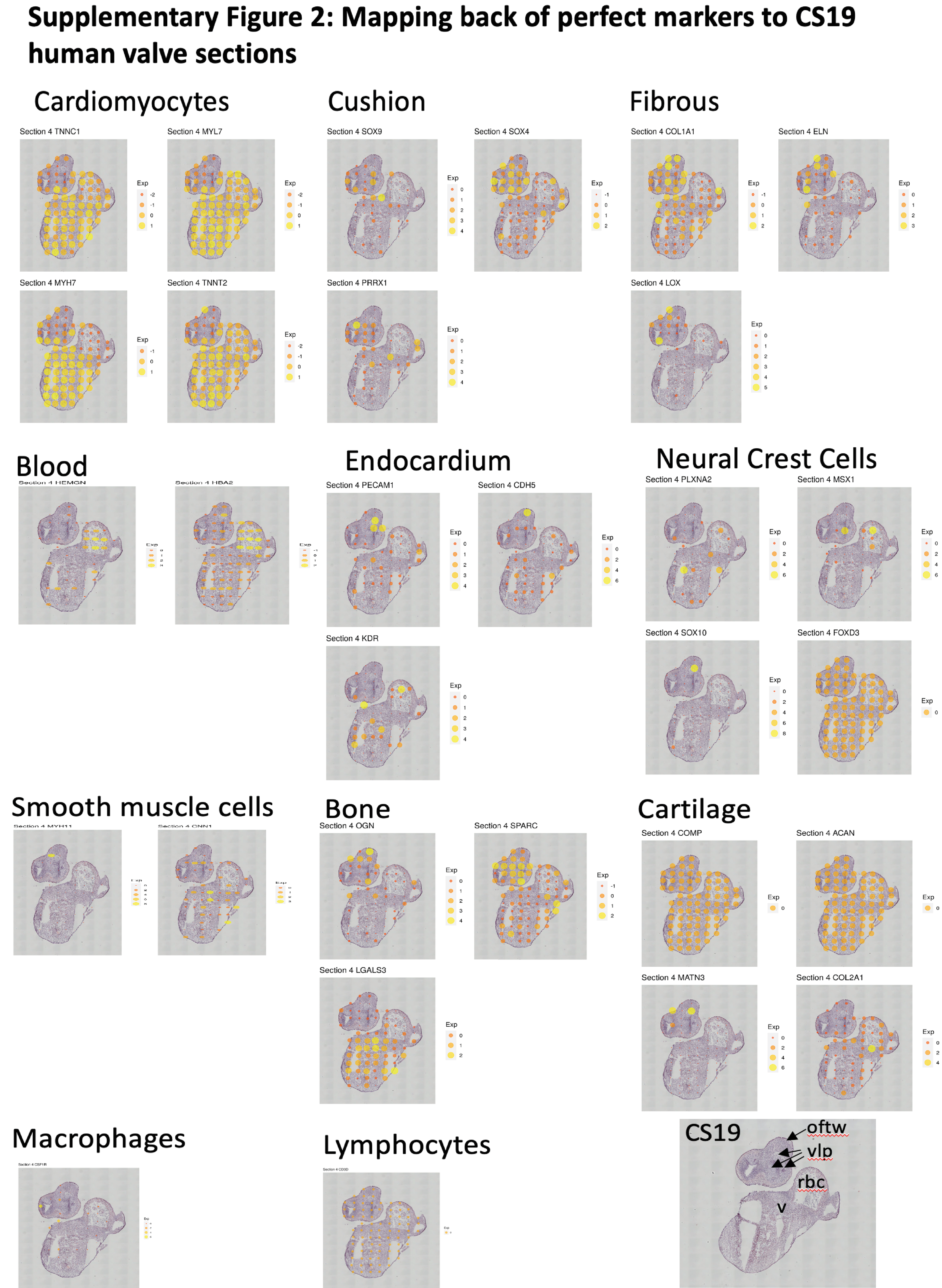

Supplement: S2 Fig — Perfect marker genes (see Fig 2) for a range of cardiac progenitor cell types (neural crest cells), differentiated cardiac cell types (cardiomyocytes, cushion tissue, fibrous tissue, endocardium, smooth muscle cells), haematopoietic cell types that are known to be found in the heart (red blood cells, lymphocytes, macrophages) and those that are known to be similar to cushion tissue (bone, cartilage), were mapped back to the CS19 human valve sections used for the ST analysis. (TIF) [file pgen.1010777.s002.tif]

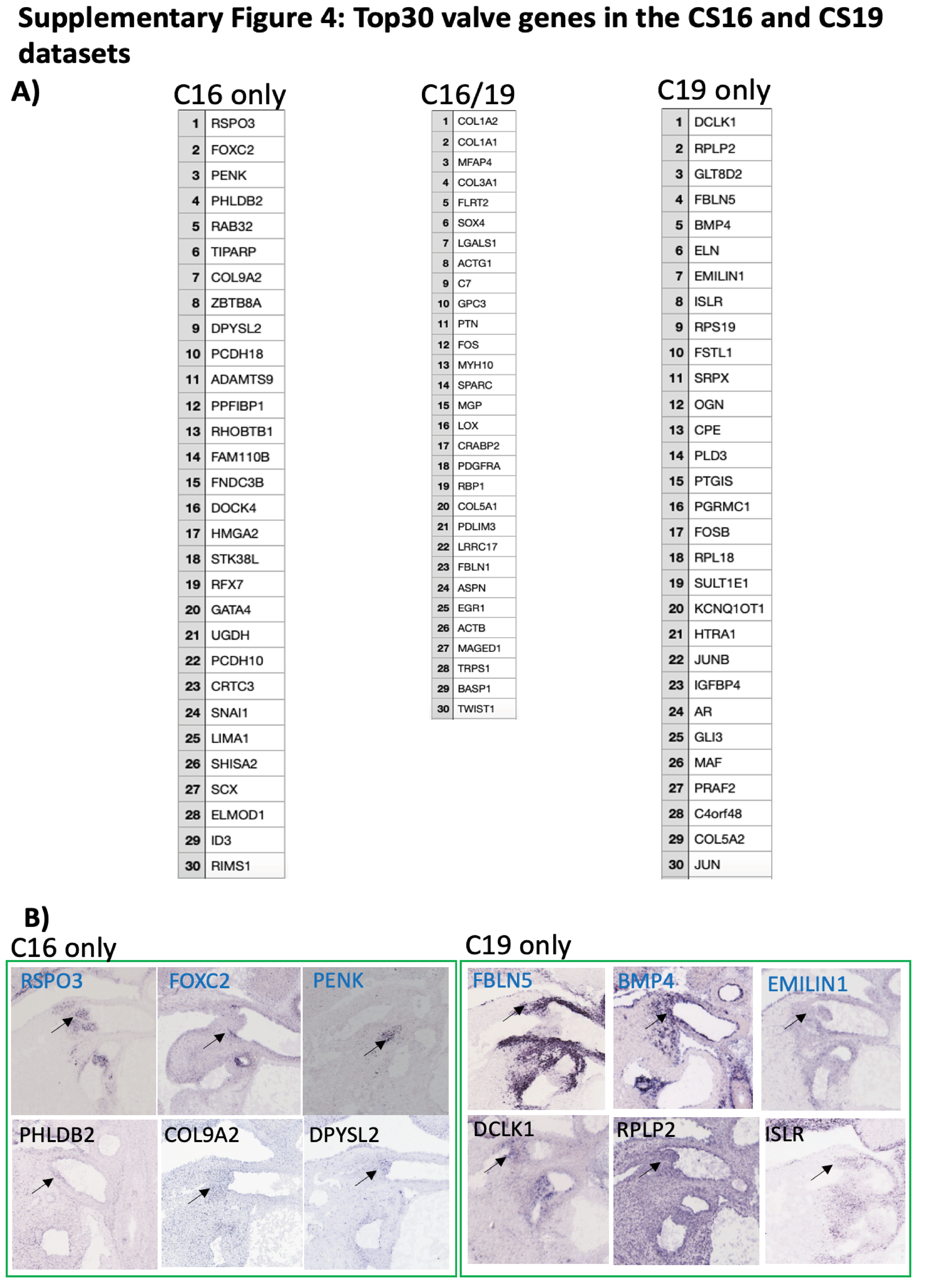

Supplement: S4 Fig — A) Top 30 DEGs in the CS16 (left list) and CS19 (right list) compared to the combined dataset. B) At both stages, GenePaint data suggested that genes already known to be expressed in the valve region (blue letters) were clearly and specifically expressed in the valve region, whereas novel genes were less strongly expressed (black letters) or were undetectable, particularly at CS16 (e.g. CS16: RAB32, TIPARP, ZBT58A; CS19:GLT8D2). (TIF) [file pgen.1010777.s004.tif]

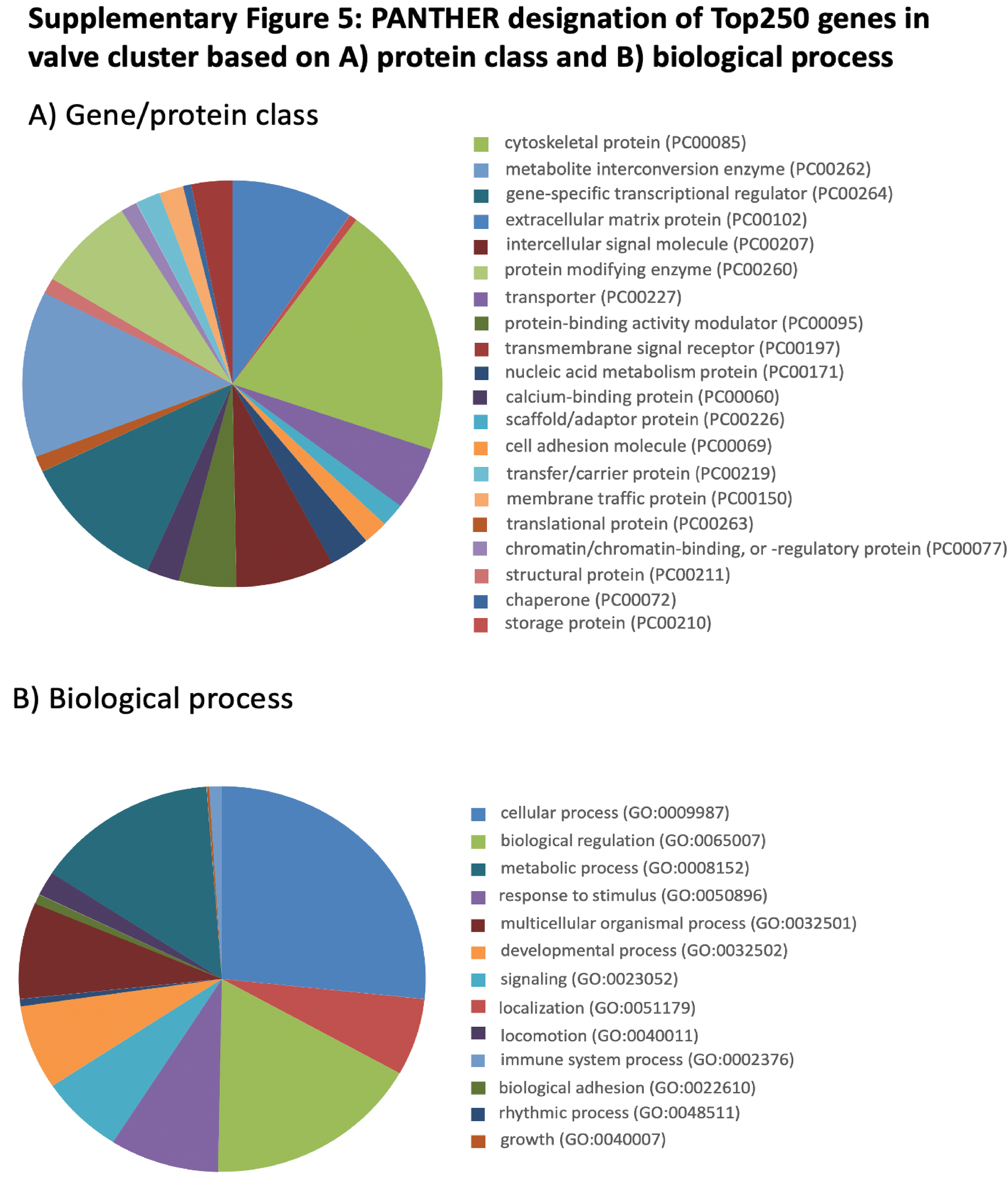

Supplement: S5 Fig — Pie charts showing A) the most common gene/protein classes and B) associated biological processes for the genes in the combined CS16/CS19 valve dataset determined using PANTHER. Gene Ontology (GO) codes are in brackets. (TIF) [file pgen.1010777.s005.tif]

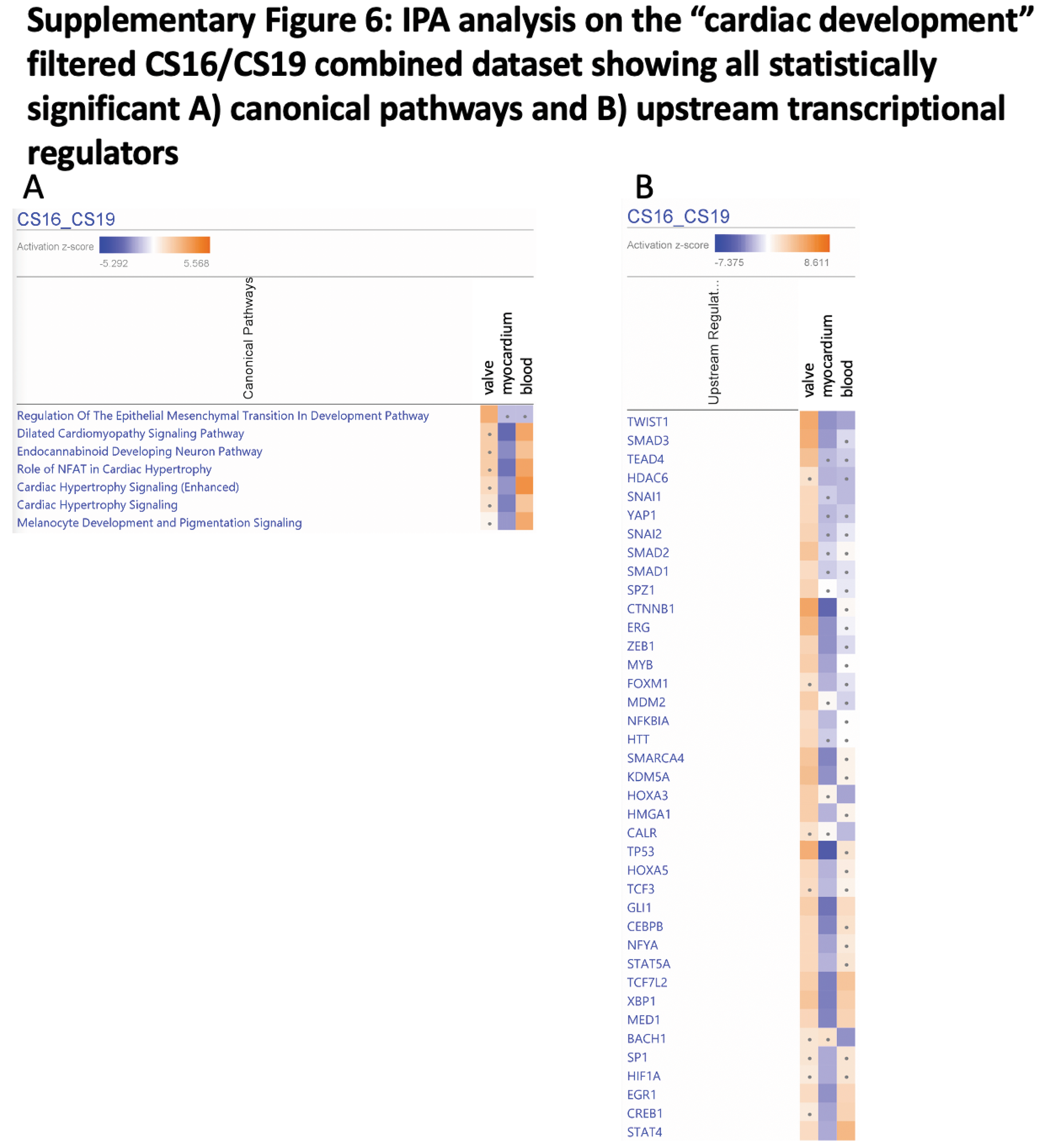

Supplement: S6 Fig — IPA analysis on the “cardiac development” filtered CS16/CS19 combined dataset showing all statistically significant A) canonical pathways and B) upstream transcriptional regulators. A) canonical pathways and B) upstream transcriptional regulators identified using Ingenuity Pathway Analysis (IPA) to scrutinise the CS16/19 combined dataset. Filtering for “cardiac” and “development”. This complements the data provided in Fig 5. (TIF) [file pgen.1010777.s006.tif]

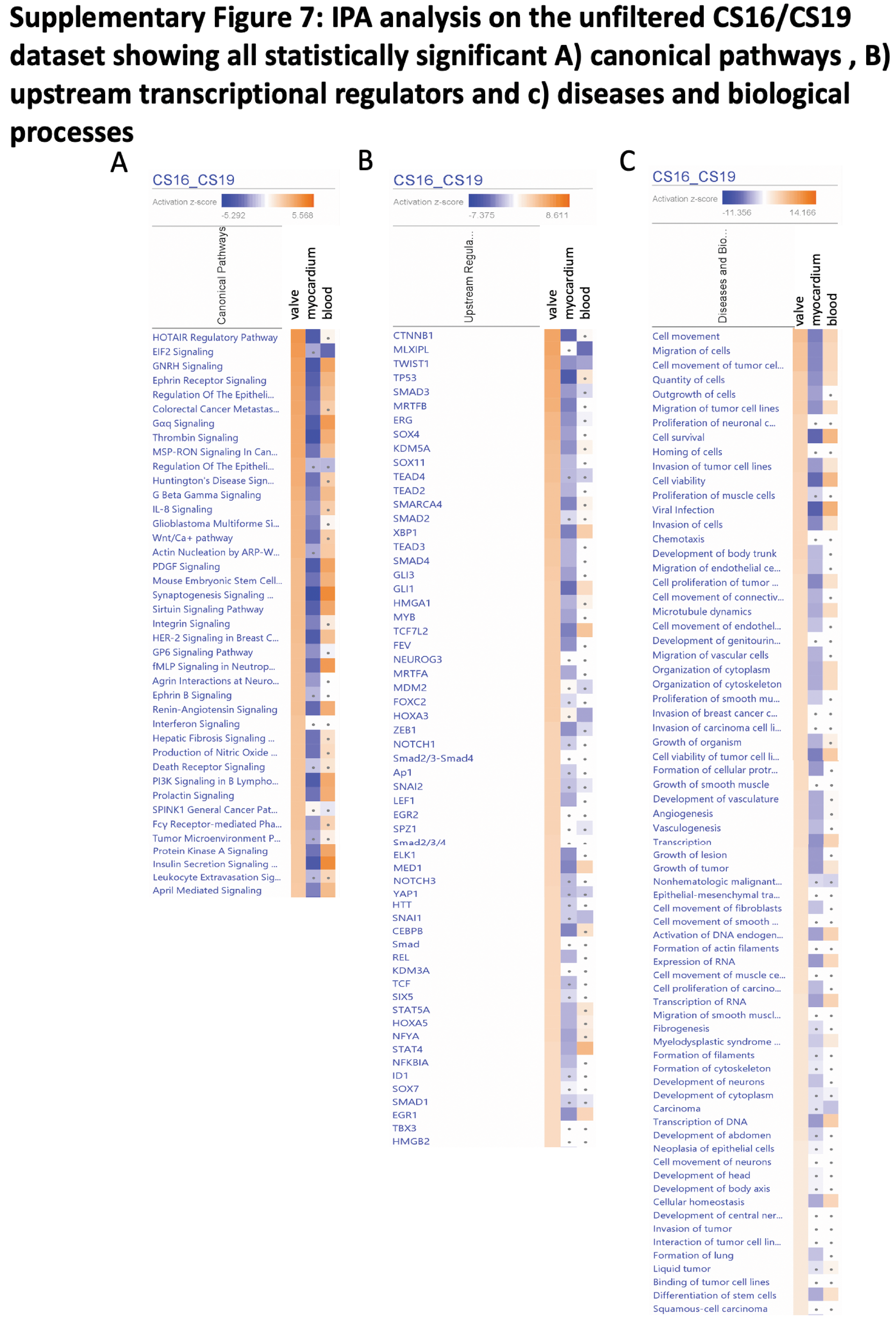

Supplement: S7 Fig — IPA analysis on the unfiltered CS16/CS19 combined dataset showing all statistically significant A) canonical pathways, B) upstream transcriptional regulators and c) diseases and biological processes. A) canonical pathways, B) upstream transcriptional regulators and c) diseases and biological processes identified using Ingenuity Pathway Analysis (IPA) to scrutinise the CS16/19 combined dataset. No filtering was applied. This complements the data provided in Fig 6. (TIF) [file pgen.1010777.s007.tif]

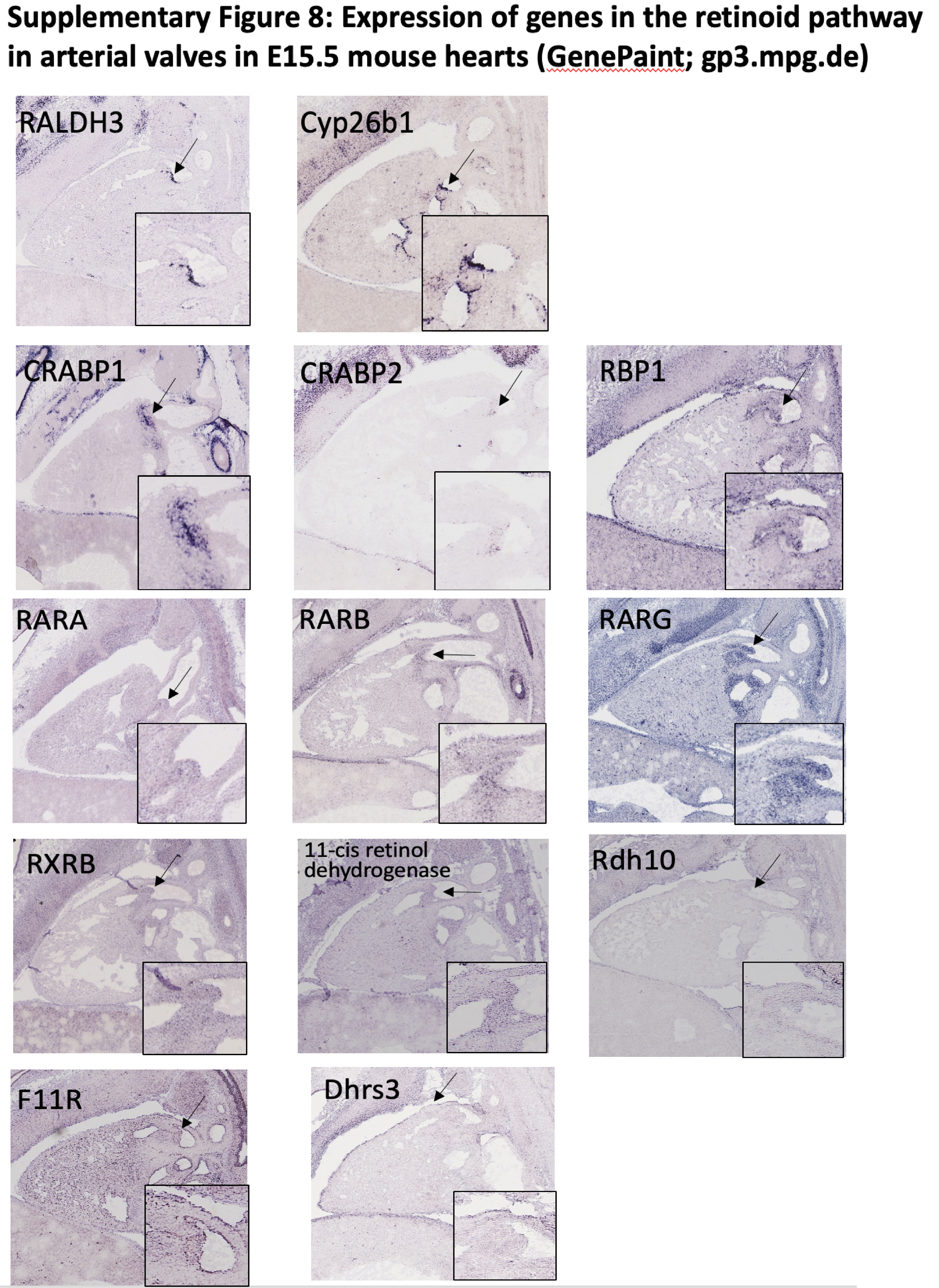

Supplement: S8 Fig — The whole embryo for each gene is shown on the left and a higher magnification image of the heart is shown on the right. In each case, the arrows point to the arterial valve. (TIF) [file pgen.1010777.s008.tif]

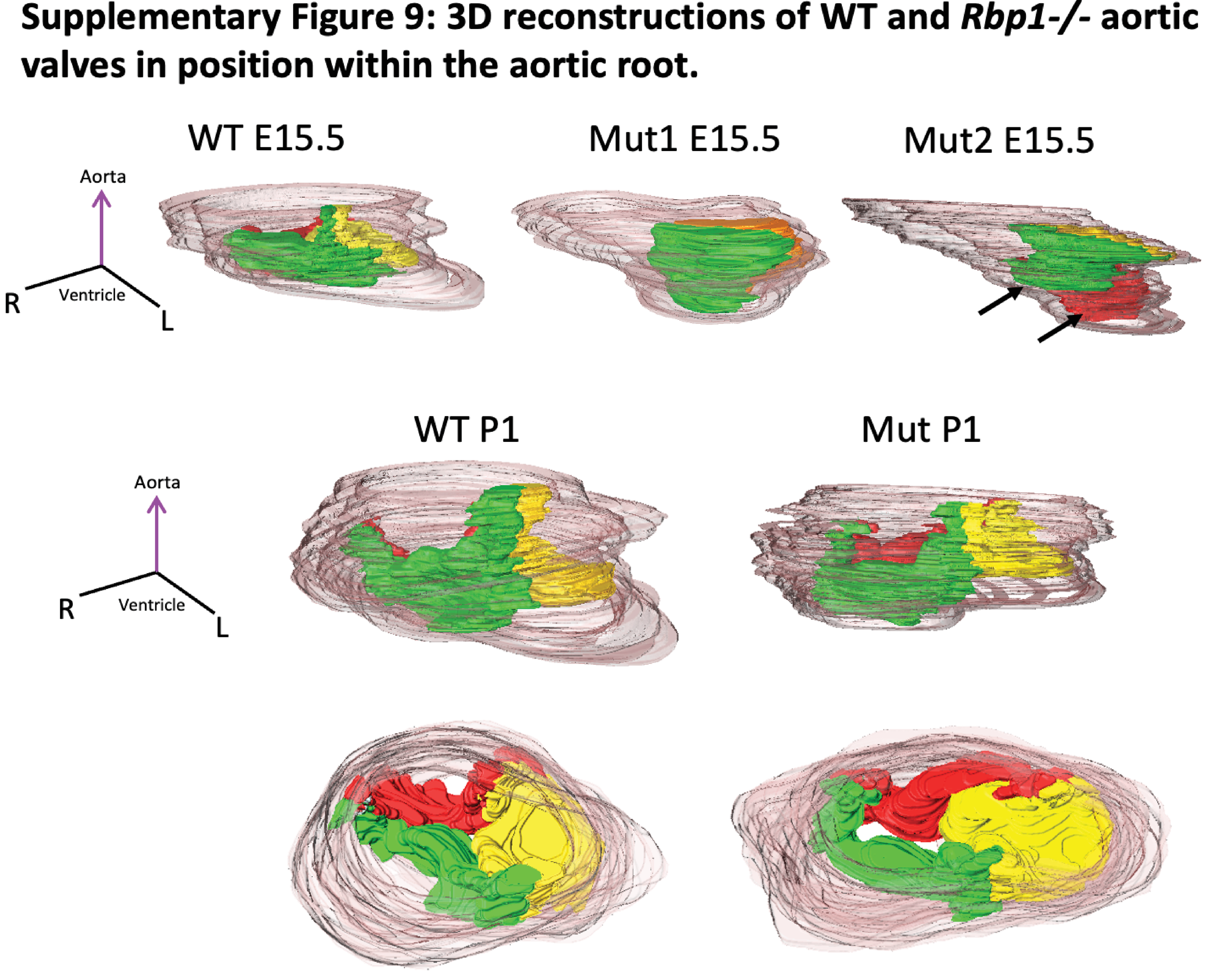

Supplement: S9 Fig — 3D reconstructions of the aortic valve of wild type and Rbp1 null mutants at E15.5 and P1 placed within the aortic root (grey). WT and Rbp1-/- mutants are matched for orientation. Red = non-coronary leaflet, yellow = left leaflet, green = right leaflet. Orange is a fused non-coronary and left leaflet. At E15.5, three leaflets were seen in the aortic valve of WT at E15.5. In comparison, two leaflets observed in a Rbp1-/- mutant (Mut 1) observed from the right side. In the other mutant (Mut 2) shown, three leaflets were observed although two were fused along the majority of their length. In this latter case, it is clear that the proximal extent of the leaflets is not the same (arrows). Three leaflets are seen in both the WT and Rbp1-/- at P1, although abnormalities in the shape and position of the leaflets are apparent. (TIF) [file pgen.1010777.s009.tif]
